# Thermo-vibrational analyses of skin tissue subjected to laser heating source in thermal therapy

**DOI:** 10.1038/s41598-021-02006-7

**Published:** 2021-11-19

**Authors:** Mina Ghanbari, Ghader Rezazadeh

**Affiliations:** 1 Mechanical Engineering Department, Urmia University of Technology, Urmia, Iran; 2 Mechanical Engineering Department, Faculty of Engineering, Urmia University, Urmia, Iran; 3 South Ural State University, Lenin prospect 76, Chelyabinsk, Russian Federation 454080

**Keywords:** Biophysics, Engineering

## Abstract

Laser-induced thermal therapy, due to its applications in various clinical treatments, has become an efficient alternative, especially for skin ablation. In this work, the two-dimensional thermomechanical response of skin tissue subjected to different types of thermal loading is investigated. Considering the thermoelastic coupling term, the two-dimensional differential equation of heat conduction in the skin tissue based on the Cattaneo–Vernotte heat conduction law is presented. The two-dimensional differential equation of the tissue displacement coupled with the two-dimensional hyperbolic heat conduction equation in the tissue is solved simultaneously to analyze the thermal and mechanical response of the skin tissue. The existence of mixed complicated boundary conditions makes the problem so complex and intricate. The Galerkin-based reduced-order model has been utilized to solve the two-sided coupled differential equations of vibration and heat transfer in the tissue with accompanying complicated boundary conditions. The effect of various types of heating sources such as thermal shock, single and repetitive pulses, repeating sequence stairs, ramp-type, and harmonic-type heating, on the thermomechanical response of the tissue is investigated. The temperature distribution in the tissue along depth and radial direction is also presented. The transient temperature and displacement response of tissue considering different relaxation times are studied, and the results are discussed in detail.

## Introduction

In modern clinical treatments, thermal therapy is considered one of the most efficient existing alternatives. In thermal therapy, electromagnetic (EM) energy, ultrasonic waves, and other devices based on thermal conduction have been used as heating sources^1^. Thermal therapy aims to modify tissue temperature in a targeted region over time to compel a desired biological response. In the majority of designed thermotherapies, thermal therapy is delivered to a target tissue volume with minimal impact on intervening or surrounding tissues. Thermal therapy emphasizes three techniques, namely, hyperthermia and thermal ablation. Hyperthermia can be categorized into long-term low-temperature hyperthermia in which the tissue temperature rises to 40–41 °C for 6–72 h and moderate-temperature hyperthermia in which tissue experiences 42–45 °C for 15–60 min. In thermal ablation, also known as high-temperature hyperthermia, the tissue temperature rises to higher than 50 °C for more than 6 min^1^. Laser-induced thermal therapy (LITT) plays a significant role in many oncology services as an alternative to conventional surgical interventions, especially for patients who are not good candidates for surgery. In other words, LITT is a percutaneous tumor-ablation procedure that delivers therapy due to the interstitially placed high-power lasers in the tumor. It delivers thermal therapy into the tumor cavity under MRI guidance, causing tumor ablation by compelling coagulation necrosis^2,3^. Reported studies show that it is also a very good option for the treatment of pediatric brain tumors^4^ and small palpable invasive breast carcinomas^5^.

Skin ablation utilizing energy-based devices has attracted increasing interest in the last few years. Skin ablation is an effective process not only for cosmetic purposes such as resurfacing, treating scars, or antiaging but also for therapeutic applications. In skin ablation, as the energy is divided into fractions, deep dermal penetration of the energy is achieved with minimal effect on the epidermis. It ensures a rapid recovery time compared with traditional ablative lasers^6^.

Several developed ablative and non‐ablative laser devices have been provided physicians with a wide palette of treatment options. In some studies, employing ablative lasers for the treatment of photoaged skin has shown efficacious results^7,8^. However, these painful procedures have several side effects, such as pigment alterations, infection, scarring, long-lasting erythema, and significant downtime. Ablative fractionated CO2 or erbium: yag lasers are commonly applied techniques. The literature on non-ablative fractional lasers (NAFLs) and ablative fractional lasers (AFLs) was reviewed by Tierney et al.^6^. This review supported the use of NAFL and AFL as an effective and safe treatment for photoaging. They showed that for the treatment of photoaging, fractionated resurfacing has important advantages over ablative laser resurfacing treatments. Bipolar radiofrequency is the other alternative method for the treatment of skin laxity, wrinkling with a low risk of scarring or persistent pigmentation^9^. It has been shown that bipolar fractionated RF treatment is considered a rapid wound healing response in which treatment intervals of at least 14 days should be recommended to allow the performance of the remodeling process. In new technologies, high-intensity focused ultrasound (HIFU) is used to treat UV-induced hyperpigmentation. This method was commonly indicated for skin laxity. Vachiramon et al. reported the efficacy and safety of high-intensity focused ultrasound for UVB-induced hyperpigmentation in human subjects^10^. In the last few years, a new technology based on thermomechanical principles has been developed to present a novel treatment modality. Fractional treatment of aging skin with Tixel was reported by Elman et al.^11^. A novel device of Tixel, which is based on thermomechanical ablation technology, synthesizes temperature control and sophisticated motion. By applying Tixel as a novel technology, average treatment pain, downtime, and erythema clearance could be improved significantly. Therefore, Tixel could be used safely for nonablative and ablative resurfacing, such as scarring, the incidence of bleeding, or postinflammatory hyperpigmentation. Sintov and Hofmann assessed the effect of Tixel on the skin penetrance of three hydrophilic molecular models^12^. In the present work, no significant damage to the skin tissue or dermal coagulation was reported. The underlying wound‐healing processes after skin ablation with thermomechanical ablation were investigated by Kokolakis et al.^13^. It was concluded that the wound‐healing process after TMA is much faster, and the recovery time minimizes significantly compared to other ablative techniques. The investigation of coagulation characteristics and ablation of a new CO laser and a high-power Tm:fiber laser were presented to assess their potential application for fractional ablation of the skin^14^. The use of a CO laser showed approximately two times larger coagulation zones than the CO_2_ laser. The practicality of using noninvasive methods for objective skin assessment was evaluated following skin rejuvenation treatment^15^. After laser treatment, considerable improvement in facial skin aesthetics was recorded in brown spots, UV spots, and pores after 3 weeks, without significant changes in the tissue at the molecular level, as assessed by micro biopsy.

Thermal therapies and physiological studies such as hyperthermia, skin and tumor ablation, cryosurgery, frostbite, skin burns, and body thermal regulation and response to environmental conditions address the temperature rise in living tissues rising from the absorbed heat of the laser plus. Therefore, temperature distribution and the process of heating transfer and in living tissues play an important role in the effectiveness of thermal therapy methods. Presenting exact mathematical modeling of the heat transfer phenomenon is difficult due to the complexity of the nature of heat transfer in living tissue. Simplifications and assumptions must be made to make the problem obedient, in addition to capturing the essential features of the process. As the determination of temperature distribution in blood perfused tissue is very significant, heat transfer in bio tissues has been studied numerously.

Several bioheat equations have been reported for modeling heat transfer in living tissue. The first bioheat equation was reported by Penne based on simplifying assumptions^16^. The assumptions of the presented model concerned four central factors: equilibrium site, blood perfusion, vascular architecture, and blood temperature. All pre-arteriole and post-venule heat transfers between blood and tissue were neglected. The flow of blood in the small capillaries was assumed to be isotropic. The effect of blood flow directionality and the local vascular geometry were also not considered in Penne’s model. Despite the shortcomings of Penne’s equation, it has enjoyed significant success in many applications, such as thermal stimulation of the whole body, cryosurgery, blood perfusion, hyperthermia therapy, and measurements. Chen and Holmes modified Penne's perfusion term, in which vascular geometry and blood flow directionality were taken into account^17^. Jiji et al. presented a three-temperature model for peripheral tissue where the temperature of arteries, veins, and tissue temperature was defined in the model for analyzing heat transfer^18^. Due to the difficulty of the three-temperature model, the three coupled deep layers were reduced to a single equation for the tissue temperature by applying simplifications^19^. The extracted differential equations for the bioheat equation in the discussed methods were all parabolic equations. They were obtained based on the classical Fourier’s law, where heat pulses are supposed to propagate at infinite speed. An extended heat conduction model was introduced by Cattaneo^20^ and Vernotte^21^. Applying Cattaneo-Vernotte (C–V) for extracting the differential heat conduction equation causes the partial differential equation to transform from parabolic to hyperbolic. In several types of research, the classical heat conduction equation has been modified to ensure finite speed pulse propagation to be compatible with physiological considerations and physical reality in a transient process^22^. To take into account the microstructure interaction effect in the heat transfer process, Tzou introduced a generalized correlation between heat flux and temperature^23,24^. As the heat conduction model requires two-phase lags of the temperature gradient and heat flux, the heat transfer model is referenced as a dual-phase lag (DPL) model. In this model, the time delay parameter was a new indicator of bioheat efficiency in living tissue.

Numerous studies have been reported on the use of the modified heat conduction equation in analyzing the temperature distribution in tissue. Analytical estimation of the temperature and thermal damage in living tissue based on a hyperbolic bioheat model was investigated by Alzahrani and Abbas^25^. The governing partial differential equation subject to laser irradiation was solved analytically in the Laplace domain. Comparing the analytical results with the existing experimental results showed the effectiveness of the mathematical model for biological heat transfer. In another work, the non-Fourier effect of laser-mediated thermal behaviors in bio tissues was investigated^26^. Optical transmission and energy deposition were obtained by applying the Monte Carlo method. The dual-phase-lag model was applied and solved by employing a three-level finite difference method. Transient heating within skin tissue due to time-dependent thermal therapy was presented in the context of a memory-dependent heat transport law^27^. The heat transport equation for this problem concerning the memory-dependent derivative was formulated in the context of the Lord-Shulman (LS) model. The effects of time-dependent moving heat source velocity and the memory-dependent derivative on the thermal injuries and temperature of skin tissues were precisely investigated. Theoretical investigation of the temperature distribution in three-dimensional biological skin tissue was reported, in which the skin tissue was irradiated by multifiber lasers ^28^. The results showed that the arrangement layout, spot size, and interval distance of the laser beams affected the obtained irradiated zone. Several types of research have been reported on the dual-phase-lag bioheat transfer model^29–31^. In one of the studies, to describe the interaction of a multipulse heat source and the skin, the dual-phase lag (DPL) bioheat transfer model and Henrique’s burn assessment model were employed^32^. The influences of the biological parameters on the temperature distribution were discussed in detail. The thermomechanical behavior taking place in instantaneously heated skin tissue via an analytical approach was explored^33^. The generalized thermoelastic model involving a dual-phase-lag model of bioheat transfer was applied in a multilayer skin structure. Variable mechanical and thermal properties with spatial location and temperature were presented. Ezzat presented analytical thermomechanical responses of viscoelastic skin tissue^34^. The influences of variable thermal conductivity and volume material properties on the heat transfer of bioheat and the mechanical heat-induced response in a human skin plane were examined.

Although several works have been presented for modeling heat transfer in bio tissues, the coupling thermoelastic term resulting from stretching or contracting of an elastic living tissue has not been considered in the heat transfer equation. Moreover, the vibration of elastic tissue in exposure to a thermal source, which is important, especially in giving thermal shock to the tissue, is rarely studied^35,36^. In this work, a two-dimensional heat conduction equation of the skin tissue coupled with the two-dimensional equation governing displacement of the tissue was presented based on a modified Fourier law. Very complicated boundary conditions appear in this problem that should be satisfied. The two-side coupled equations of heat transfer and tissue movement are solved simultaneously to analyze the transient temperature and vibrating response of the tissue. The effect of several types of laser heat sources on the response of the tissue is investigated in more detail. The effect of thermoelastic terms on the temperature response of the tissue is also studied.

## Governing equations

**Figure 1:**
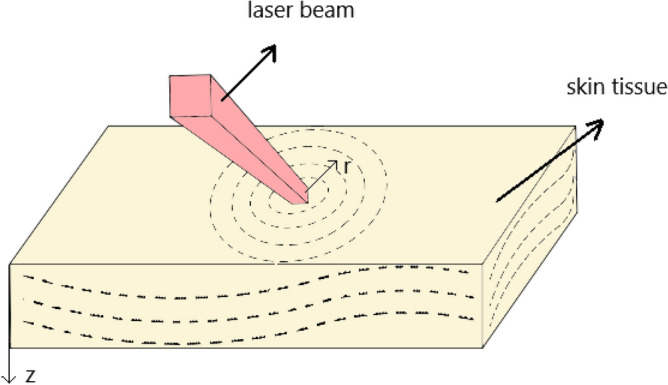
Thermoelastic vibration of the skin tissue in the presence of laser irradiation.

Figure 1 shows the biological skin tissue exposed to laser heating in cylindrical coordinates. When biological tissue receives laser irradiation, it will experience localized heating, optical energy absorption, and thermal expansion. By supposing an axisymmetric temperature distribution in the skin tissue, a two-dimensional distribution is considered along with the r- and z-directions in this study. Tissues can also vibrate along with the radial (r) and the skin depth directions (z) by absorbing optical energy. The two-sided coupled governing equations of the two-dimensional heat conduction and two-dimensional vibration of the elastic media in cylindrical coordinates are extracted. They are solved simultaneously to obtain the temperature response of the bio tissue exposed to laser heat. It is assumed that the radius of the laser beam is so small that the laser beam is assumed to be a localized heating source at r = 0.

According to the anatomical structure of skin tissue, it is generally considered as a multilayered structure consist of epidermis, dermis, fat layers. Different layers of skin tissue have different nonlinear thermomechanical behaviors. Moreover, the physical properties of each layer are also age-dependent and can vary in different ages of humans^37,38^. Few types of research have been reported the nonlinear elastic behavior to identify the mechanical parameters of human skin. Delalleau et al. defined an identification method related to a new numerical model to account for the nonlinear behavior of human skin in vivo^39^. In that work, a relationship between mechanical and physiological aspects was first presented. The orientation of the dermis collagen fibers in the direction of the stress during an experiment made changes in the elasticity of the skin and showed that skin tissue becomes stiffer. This leads to a three-phase behavior law and validates the nonlinear mechanical approach. In another research Finite element modeling of human skin was introduced using an anisotropic, nonlinear elastic constitutive model^40^. It was shown that the nonlinear mechanical stress–strain response of skin is originated from the collagen network in the skin. It was hypothesized that the force-stretch response of collagen is governed by the entropic of long-chain molecules. A constitutive model derived from the statistical mechanics of long-chain molecules, corresponding to the " collagen network in the skin", demonstrated the mechanical response of the skin. In this work, for simplicity, a linear single-layer skin model is used where the skin is treated as a single homogeneous layer with constant thermal and physical properties.

### Two-dimensional displacement equation of the tissue

The balanced equation of an elastic media is expressed as^41–43^:

1 $${\mathfrak{S}}_{ij,j}+\rho {\fancyscript{b}}_{i}=\rho {\ddot{\mathcal{U}}}_{i}$$

where $\rho$ is the tissue density, ${\fancyscript{b}}_{i}$ is the body force per unit volume of the tissue, ${\mathfrak{S}}_{ij}$ refers to stress components and ${\mathcal{U}}_{i}$ denotes the tissue displacement components. The balance equation in cylindrical coordinates in the r- and z-directions is expressed as ^41–43^:

2 $$\frac{\partial {\mathfrak{S}}_{rr}}{\partial r}+\frac{1}{r}\frac{\partial {\mathfrak{S}}_{r\theta }}{\partial \theta }+\frac{\partial {\mathfrak{S}}_{rz}}{\partial z}+\frac{1}{r}\left({\mathfrak{S}}_{rr}-{\mathfrak{S}}_{\theta \theta }\right)+\rho {\fancyscript{b}}_{r}=\rho \frac{{\partial }^{2}\fancyscript{u}}{\partial {\fancyscript{t}}^{2}}$$

3 $$\frac{\partial {\mathfrak{S}}_{rz}}{\partial r}+\frac{1}{r}\frac{\partial {\mathfrak{S}}_{\theta z}}{\partial \theta }+\frac{\partial {\mathfrak{S}}_{zz}}{\partial z}+\frac{1}{r}{\mathfrak{S}}_{rz}+\rho {\fancyscript{b}}_{z}=\rho \frac{{\partial }^{2}\fancyscript{v}}{\partial {\fancyscript{t}}^{2}}$$

4 $$\frac{\partial {\mathfrak{S}}_{r\theta }}{\partial r}+\frac{1}{r}\frac{\partial {\mathfrak{S}}_{\theta \theta }}{\partial \theta }+\frac{\partial {\mathfrak{S}}_{\theta z}}{\partial z}+\frac{2}{r}{\sigma }_{r\theta }+\rho {\fancyscript{b}}_{r}=\rho \frac{{\partial }^{2}\fancyscript{w}}{\partial {\fancyscript{t}}^{2}}$$

where $\fancyscript{u}$ and $\fancyscript{v}$ and $\fancyscript{w}$ are displacement components of tissue displacement in the r-, z-, and $\theta$-directions. The constitutive equation of an elastic media in the presence of the temperature distribution is defined as:

5 $${\mathfrak{S}}_{ij}=\lambda {\varepsilon }_{kk}{\delta }_{ij}+2\mu {\varepsilon }_{ij}-\mathfrak{B}\Delta \mathcal{T}{\delta }_{ij}$$

In Eq. (5), ${\delta }_{ij}$ is the Kronecker delta, and $\mathcal{T}$ is the temperature. $\lambda$ and $\mu$ are the Lame constants related to the modulus of elasticity $E$ and Poisson’s ratio $\nu$ as^41^:

6 $$\lambda =\frac{Ev}{(1+v)(1-2v)}; \mu =\frac{E}{2(1+v)}$$

where, ${\varepsilon }_{ij}=\frac{1}{2}\left({u}_{i,j}+{u}_{j,i}\right)$ is the strain tensor. In relation (5), $\mathfrak{B}$ is defined as:

7 $$\lambda =\frac{E{\alpha }_{\mathcal{T}}}{1-2\upsilon }$$

where ${\alpha }_{T}$ is the thermal expansion, $E$ is the elasticity modulus of the tissue and $\upsilon$ is Poisson’s ratio.

The components of the strain tensor in cylindrical coordinates are expressed as^41^:

8 $$\begin{aligned}&{\mathcal{E}}_{rr}=\frac{\partial \fancyscript{u}}{\partial r};{\mathcal{E}}_{\theta \theta }=\frac{1}{r}\left(\frac{\partial \fancyscript{w}}{\partial \theta }+\fancyscript{u}\right); {\mathcal{E}}_{zz}=\frac{\partial \fancyscript{v}}{\partial z}; {\mathcal{E}}_{r\theta }=\frac{1}{2}\left(\frac{1}{r}\frac{\partial \fancyscript{u}}{\partial \theta }+\frac{\partial \fancyscript{w}}{\partial r}-\frac{\fancyscript{w}}{r}\right);\\&{\mathcal{E}}_{\theta z}=\frac{1}{2}\left(\frac{\partial \fancyscript{w}}{\partial z}+\frac{1}{r}\frac{\partial \fancyscript{v}}{\partial \theta }\right); {\mathcal{E}}_{zr}=\frac{1}{2}\left(\frac{\partial \fancyscript{u}}{\partial z}+\frac{\partial \fancyscript{v}}{\partial r}\right)\end{aligned}$$

Substituting strain components (8) into constitutive equation (5), the components of the stress tensor in cylindrical coordinates can be expressed as:

9 $$\begin{aligned}&{\mathfrak{S}}_{rr}= \lambda \left(\frac{\partial \fancyscript{u}}{\partial r}+\frac{1}{r}\left(\frac{\partial \fancyscript{w}}{\partial \theta }+\fancyscript{u}\right)+\frac{\partial \fancyscript{v}}{\partial z}\right)+2\mu \frac{\partial \fancyscript{u}}{\partial r}-\mathfrak{B}\left(\mathcal{T}\left(r,z,t\right)-{\mathcal{T}}_{0}\right);\\&{\mathfrak{S}}_{r\theta }=\mu \left(\frac{1}{r}\frac{\partial \fancyscript{u}}{\partial \theta }+\frac{\partial \fancyscript{w}}{\partial r}-\frac{\fancyscript{w}}{r}\right);{\mathfrak{S}}_{rz}=\mu \left(\frac{\partial \fancyscript{u}}{\partial z}+\frac{\partial \fancyscript{v}}{\partial r}\right);\\&{\mathfrak{S}}_{\theta \theta }= \lambda \left(\frac{\partial \fancyscript{u}}{\partial r}+\frac{1}{r}\left(\frac{\partial \fancyscript{w}}{\partial \theta }+\fancyscript{u}\right)+\frac{\partial \fancyscript{v}}{\partial z}\right)+2\mu \left(\frac{1}{r}\left(\frac{\partial \fancyscript{w}}{\partial \theta }+\fancyscript{u}\right)\right)-\mathfrak{B}\left(T\left(r,z,t\right)-{T}_{0}\right);\\&{\mathfrak{S}}_{\theta z}=2\mu \left(\frac{\partial \fancyscript{w}}{\partial z}+\frac{1}{r}\frac{\partial \fancyscript{v}}{\partial \theta }\right);\\& {\mathfrak{S}}_{zz}= \lambda \left(\frac{\partial \fancyscript{u}}{\partial r}+\frac{1}{r}\left(\frac{\partial \fancyscript{w}}{\partial \theta }+\fancyscript{u}\right)+\frac{\partial \fancyscript{v}}{\partial z}\right)+2\mu \left(\frac{\partial \fancyscript{v}}{\partial z}\right)-\mathfrak{B}\left(\mathcal{T}\left(r,z,t\right)-{\mathcal{T}}_{0}\right)\end{aligned}$$

${T}_{0}$ is the reference temperature.

Substituting relation (9) into balance equations (2) and (3), the two-dimensional vibration of tissue in the r- and z-directions takes the following form:

10 $$\left(\lambda +2\mu \right)\left(\frac{{\partial }^{2}\fancyscript{u}}{\partial {\fancyscript{r}}^{2}}+\frac{1}{\fancyscript{r}}\left(\frac{\partial \fancyscript{u}}{\partial \fancyscript{r}}\right)-\left(\frac{\fancyscript{u}}{{\fancyscript{r}}^{2}}\right)\right)+\mu \left(\frac{{\partial }^{2}\fancyscript{u}}{\partial {\fancyscript{z}}^{2}}\right)+\left(\lambda +\mu \right)\left(\frac{{\partial }^{2}\fancyscript{v}}{\partial \fancyscript{z}\partial \fancyscript{r}}\right)-\mathfrak{B}\frac{\partial \mathcal{T}}{\partial \fancyscript{r}}= \rho \frac{{\partial }^{2}\fancyscript{u}}{\partial {\fancyscript{t}}^{2}}+\fancyscript{c}\frac{\partial \fancyscript{u}}{\partial \fancyscript{t}}$$

11 $$\left(\lambda +2\mu \right)\left(\frac{{\partial }^{2}\fancyscript{v}}{\partial {\fancyscript{z}}^{2}}\right)+\mu \left(\frac{{\partial }^{2}\fancyscript{v}}{\partial {\fancyscript{r}}^{2}}+\frac{1}{\fancyscript{r}}\left(\frac{\partial \fancyscript{v}}{\partial \fancyscript{r}}\right)\right)+\left(\lambda +\mu \right)\left(\frac{{\partial }^{2}\fancyscript{u}}{\partial \fancyscript{r}\partial \fancyscript{z}}+\frac{1}{r}\left(\frac{\partial \fancyscript{u}}{\partial \fancyscript{z}}\right)\right)-\mathfrak{B}\frac{\partial \mathcal{T}}{\partial \fancyscript{z}}=\rho \frac{{\partial }^{2}\fancyscript{v}}{\partial {\fancyscript{t}}^{2}}+\fancyscript{c}\frac{\partial \fancyscript{v}}{\partial \fancyscript{t}}$$

### Two-dimensional hyperbolic heat transfer equation

Due to the importance of temperature distribution in bio tissue in many medical therapies, such as brain tumor invasive breast carcinomas, hyperthermia, frostbite, and skin burns, predicting the accurate temperature response of bio tissue to laser irradiation seems to be very important. Due to the complexity of the nature of heat transfer in living tissue, presenting a mathematical model for heat transfer needs logical simplifications. Several simplifications and assumptions must be made to make the problem tractable while capturing the essential features of the process. The local form of the energy balance equation in index notation is as follows:

12 $${\int }_{V}\left(\dot{u}-{\mathfrak{S}}_{ij}{\dot{\varepsilon }}_{ij}-\mathcal{Q}+{q}_{i,i}\right)dV=0$$

where $\sigma$ is the stress, $\varepsilon$ is the strain, $u$ is the specific internal energy per unit volume, $Q$ is known as the heat supply and denotes the rate at which heat per unit volume is produced by internal sources, and $V$ is the volume. The rate at which heat is conducted into the body per unit area per unit time across the element of the surface is called the heat flux vector and is represented by the symbol ${q}_{i}$. Fourier’s law states that the time rate of heat transfer through a material is proportional to the negative gradient of temperature and to the area at right angles through which the heat flows. It specifies a linear relationship between the temperature gradient and heat flux and is expressed as:

13 $${q}_{i}=-k{\mathcal{T}}_{,i}$$

In Eq. (13), $\mathcal{T}$ is temperature and $k$ is the thermal conductivity $\left(\frac{\mathrm{W}}{\mathrm{m}\,\mathrm{k}}\right)$. In Fourier’s law, the diffusion of heat gives rise to infinite speeds of heat propagation, which is incompatible with physical reality and physiological considerations in a transient process. Rapid thermal energy deposition is indicated in the medium, i.e., the occurrence of any local temperature disturbance leads to an immediate perturbation in temperature at each point in the medium. Applying Fourier’s law in the energy equation results in Pennes’ bioheat equation, which is commonly used for modeling heat transfer in biological systems. Much attention has been assigned to developing the classical heat conduction equation to ensure finite-speed pulse propagation. The generalized Fourier’s law is called the Cattaneo–Vernotte heat conduction law. Utilizing it, the governing partial differential equation is transformed from parabolic to hyperbolic type. The constitutive equation of the Cattaneo–Vernotte model is characterized as follows:

14 $${q}_{i}+\tau {\dot{q}}_{i}=k{\mathcal{T}}_{,i}$$

$\tau$ is the relaxation time, assumed to be a non-negative constant.

Combining equations (12) and (14) results in the following heat conduction equation^46^:

15 $${\mathcal{T}}_{,ii}+\mathcal{Q}+\tau \dot{\mathcal{Q}}=\rho c\dot{\mathcal{T}}+\rho c\tau \ddot{\mathcal{T}}$$

In Eq. (15), $c$ is the specific heat capacity of the tissue. The thermoelastic coupling term in the heat conduction equation is so small and usually neglected in practice ^44^. It is important in many applications, such as thermal shocks^45,46^, ultrafast laser heating in the thermal processing of materials^47,48^, modeling of the vibration of resonant microelectromechanical systems (MEMS)^49–52^, and dynamic crack propagation^53^. Therefore, the thermoelastic effect should be taken into account in the mentioned application. By considering the thermoelastic effect in Eq. (15), it is modified to^46^:

16 $$k{\mathcal{T}}_{,ii}+\mathcal{Q}+\tau \dot{\mathcal{Q}}=\rho c\dot{\mathcal{T}}+\rho c\tau \ddot{\mathcal{T}}+\mathfrak{B}{\mathcal{T}}_{0}\left({\dot{\varepsilon }}_{kk}+\tau {\ddot{\varepsilon }}_{kk}\right)$$

$Q$ is the volumetric heat generated in the tissue by blood perfusion, metabolism, and laser pulses as:

17 $$\mathcal{Q}={\mathcal{Q}}_{\mathcal{L}}+{\mathcal{Q}}_{\mathcal{B}}+{\mathcal{Q}}_{\mathcal{M}}$$

In Eq. (17), ${\mathcal{Q}}_{\mathcal{L}}$ is the volumetric heat generated from a laser heat source^25^:

18 $${\mathcal{Q}}_{\mathcal{L}}\left(z,t\right)={\fancyscript{f}}_{1}(z){\fancyscript{f}}_{2}(t)$$

where $\fancyscript{f}\left(z\right)={I}_{0}{\mu }_{a}\left[{\mathfrak{O}}_{1}{e}^{-\frac{{e}_{1}z}{\fancyscript{h}}}-{\mathfrak{O}}_{2}{e}^{-\frac{{e}_{2}z}{\fancyscript{h}}}\right]$. ${I}_{0}$ is the laser intensity, ${\mu }_{a}$ is the absorption coefficient, and ${e}_{1}$, ${e}_{2}$, ${\mathfrak{O}}_{1}$ and ${\mathfrak{O}}_{2}$ are functions of diffuse reflectance $\left({R}_{d}\right)$ and are presented by Gardner et al.^25^.${\fancyscript{f}}_{2}(t)$ is a time-dependent function and can be considered in any form as a repetitive pulse function, step function, harmonic function, and so on. $\delta$ is the penetration depth and is characterized as follows^25^:

19 $$\delta =\frac{1}{\sqrt{3{\mu }_{a}\left({\mu }_{a}+{\mu }_{s}\left(1-\fancyscript{g}\right)\right)}}$$

$\fancyscript{g}$ is the anisotropy factor, and ${\mu }_{s}$ is the scattering coefficient.

${\mathcal{Q}}_{\mathcal{B}}$ in relation (17) is the volumetric heat generated by blood perfusion and is defined as:

20 $${\mathcal{Q}}_{\mathcal{B}}={\rho }_{b}{c}_{b}{\omega }_{b}({\mathcal{T}}_{b}-\mathcal{T})$$

where ${\rho }_{b}$ and ${c}_{b}$ are the density and the specific heat capacity of the blood, ${\omega }_{b}$ is the blood perfusion, and ${\mathcal{T}}_{b}$ is the artery temperature.${\mathcal{Q}}_{\mathcal{M}}$ is the heat generated by the metabolic process due to various physiological processes occurring in the rest of the body and is neglected in this study.

Substituting relations (8) and (17) in relation (16), the two-dimensional hyperbolic heat conduction equation is expressed as:

21 $$\begin{aligned}&k\left(\frac{{\partial }^{2}\mathcal{T}}{\partial {\fancyscript{r}}^{2}}+\frac{1}{\fancyscript{r}}\frac{\partial \mathcal{T}}{\partial \fancyscript{r}}+\frac{{\partial }^{2}\mathcal{T}}{\partial {\fancyscript{z}}^{2}}\right)+{\rho }_{b}{c}_{b}{\omega }_{b}{\mathcal{T}}_{b}-{\rho }_{b}{c}_{b}{\omega }_{b}\mathcal{T}\mathcal{T}-\tau {\rho }_{b}{c}_{b}{\omega }_{b}\frac{\partial \mathcal{T}}{\partial \fancyscript{t}}+{\mathcal{Q}}_{\mathcal{L}}\left(z,t\right)+\tau {\dot{\mathcal{Q}}}_{\mathcal{L}}\left(z,t\right)\\&=\rho c\frac{\partial \mathcal{T}}{\partial \fancyscript{t}}+\rho c\tau \frac{{\partial }^{2}\mathcal{T}}{\partial {\fancyscript{t}}^{2}}+\mathfrak{B}{\mathcal{T}}_{0}\left(\frac{{\partial }^{2}\fancyscript{u}}{\partial \fancyscript{t}\partial \fancyscript{r}}+\frac{1}{\fancyscript{r}}\frac{\partial \fancyscript{u}}{\partial \fancyscript{t}}+\frac{{\partial }^{2}\fancyscript{v}}{\partial \fancyscript{t}\partial \fancyscript{z}}\right)\\&\quad+\mathfrak{B}{\mathcal{T}}_{0}\tau \left(\frac{{\partial }^{3}\fancyscript{u}}{\partial {\fancyscript{t}}^{2}\partial \fancyscript{r}}+\frac{1}{r}\frac{{\partial }^{2}\fancyscript{u}}{\partial {\fancyscript{t}}^{2}}+\frac{{\partial }^{3}\fancyscript{v}}{\partial {\fancyscript{t}}^{2}\partial \fancyscript{z}}\right)\end{aligned}$$

By considering the following nondimensional parameters:

22 $$\theta =\frac{\mathcal{T}-{\mathcal{T}}_{0}}{{\mathcal{T}}_{0}};{\theta }_{b}=\frac{{\mathcal{T}}_{b}-{\mathcal{T}}_{0}}{{\mathcal{T}}_{0}};\widehat{r}=\frac{\fancyscript{r}}{\delta };\widehat{u}=\frac{\fancyscript{u}}{\delta };\widehat{t}=\frac{\fancyscript{t}}{{t}^{*}};{t}^{*}=\frac{{\delta }^{2}}{\alpha };\widehat{z}=\frac{\fancyscript{z}}{\delta };\widehat{v}=\frac{\fancyscript{v}}{\delta };$$

where $\alpha =k/\rho c$ $\left({\mathrm{m}}^{2}/\mathrm{s}\right)$ is the heat diffusivity of the tissue. Substituting the parameters (22) into Eqs. (10–11), and (21) yields:

23 $$\frac{{\partial }^{2}\widehat{u}}{\partial {\widehat{t}}^{2}}-{\mathfrak{M}}_{1}\frac{\partial \widehat{u}}{\partial \widehat{t}}-{\mathfrak{M}}_{2}\left(\frac{{\partial }^{2}\widehat{u}}{\partial {\widehat{r}}^{2}}\right)-{\mathfrak{M}}_{2}\frac{1}{\widehat{r}}\left(\frac{\partial \widehat{u}}{\partial \widehat{r}}\right)+{\mathfrak{M}}_{2}\left(\frac{\widehat{u}}{{\widehat{r}}^{2}}\right)-{\mathfrak{M}}_{3}\left(\frac{{\partial }^{2}\widehat{u}}{\partial {\widehat{z}}^{2}}\right)-{\mathfrak{M}}_{4}\left(\frac{{\partial }^{2}\widehat{v}}{\partial \widehat{z}\partial \widehat{r}}\right)+{\mathfrak{M}}_{5}\frac{\partial \theta }{\partial \widehat{r}}=0$$

24 $$\frac{{\partial }^{2}\widehat{v}}{\partial {\widehat{t}}^{2}}-{\mathfrak{M}}_{1}\frac{\partial \widehat{v}}{\partial \widehat{t}}-{\mathfrak{M}}_{2}\left(\frac{{\partial }^{2}\widehat{v}}{\partial {\widehat{z}}^{2}}\right)-{\mathfrak{M}}_{3}\left(\frac{{\partial }^{2}\widehat{v}}{\partial {\widehat{r}}^{2}}\right)-{\mathfrak{M}}_{3}\left(\frac{1}{\widehat{r}}\left(\frac{\partial \widehat{v}}{\partial \widehat{r}}\right)\right)-{\mathfrak{M}}_{4}\left(\frac{{\partial }^{2}\widehat{u}}{\partial \widehat{r}\partial \widehat{z}}\right)-{\mathfrak{M}}_{4}\left(\frac{1}{\widehat{r}}\left(\frac{\partial \widehat{u}}{\partial \widehat{z}}\right)\right)+{\mathfrak{M}}_{5}\frac{\partial \theta }{\partial \widehat{z}}=0$$

25 $$\begin{aligned}\frac{{\partial }^{2}\theta }{\partial {\widehat{t}}^{2}}+{\mathfrak{N}}_{1}\left(\frac{{\partial }^{3}\widehat{u}}{\partial {\widehat{t}}^{2}\partial \widehat{r}}\right)+{\mathfrak{N}}_{1}\left(\frac{1}{\widehat{r}}\frac{{\partial }^{2}\widehat{u}}{\partial {\widehat{t}}^{2}}\right)+{\mathfrak{N}}_{1}\left(\frac{{\partial }^{3}\widehat{v}}{\partial {\widehat{t}}^{2}\partial \widehat{z}}\right)+{\mathfrak{N}}_{2}\frac{\partial \theta }{\partial \widehat{t}}+{\mathfrak{N}}_{3}\left(\frac{{\partial }^{2}\widehat{u}}{\partial \widehat{t}\partial \widehat{r}}\right)\\&\quad+{\mathfrak{N}}_{3}\left(\frac{1}{\widehat{r}}\frac{\partial \widehat{u}}{\partial \widehat{t}}\right)+{\mathfrak{N}}_{3}\left(\frac{{\partial }^{2}\widehat{v}}{\partial \widehat{t}\partial \widehat{z}}\right)-{\mathfrak{N}}_{4}\left(\frac{{\partial }^{2}\theta }{\partial {\widehat{r}}^{2}}\right)-{\mathfrak{N}}_{7}\left(\frac{1}{\widehat{r}}\frac{\partial \theta }{\partial \widehat{r}}\right)\\&\quad-{\mathfrak{N}}_{7}\left(\frac{{\partial }^{2}\theta }{\partial {\widehat{z}}^{2}}\right)-{\varrho }_{4}{\theta }_{b}+{\varrho }_{4}\theta \left(\widehat{r},\widehat{z},\widehat{t}\right)-{\mathfrak{N}}_{5}{\widehat{\mathcal{Q}}}_{\mathcal{L}}\left(\widehat{r},\widehat{z},\widehat{t}\right)-{\mathfrak{N}}_{6}\dot{{\widehat{\mathcal{Q}}}_{\mathcal{L}}}\left(\widehat{r},\widehat{z},\widehat{t}\right)=0\end{aligned}$$

with the following coefficients:

26 $$\begin{aligned}&{\mathfrak{M}}_{1}=\frac{\fancyscript{c}{\delta }^{2}}{\rho \alpha };{\mathfrak{M}}_{2}=\frac{\left(\lambda +2\mu \right){\delta }^{2}}{\rho {\alpha }^{2}};{\mathfrak{M}}_{3}=\frac{\mu {\delta }^{2}}{\rho {\alpha }^{2}};{\mathfrak{M}}_{4}=\frac{\left(\lambda +\mu \right){\delta }^{2}}{\rho {\alpha }^{2}};{\mathfrak{M}}_{5}=\frac{\mathfrak{B}{T}_{0}{\delta }^{2}}{\rho {\alpha }^{2}};\\ &{\mathfrak{N}}_{1}=\frac{\beta }{\rho c} ;{\mathfrak{N}}_{2}=\left(\frac{{t}^{*}}{\tau }+\frac{{\rho }_{b}{c}_{b}{G}_{b}{t}^{*}}{\rho c}\right);{\mathfrak{N}}_{3}=\left(\frac{\beta {t}^{*}}{\rho c\tau }\right); {\mathfrak{N}}_{4}=\frac{{\rho }_{b}{c}_{b}{G}_{b}{{t}^{*}}^{2}}{\rho c\tau }\\&{\mathfrak{N}}_{5}=\frac{{{t}^{*}}^{2}}{\rho c\tau {T}_{0}};{\mathfrak{N}}_{6}=\frac{{{t}^{*}}^{2 }}{\rho c{T}_{0}}; {\mathfrak{N}}_{7}=\frac{{\delta }^{2}}{\tau \alpha } \end{aligned}$$

In Eq. (25) the source term is expressed as:

27 $${\widehat{\mathcal{Q}}}_{\mathcal{L}}\left(\widehat{z},\widehat{t}\right)={\fancyscript{f}}_{1}\left(\overline{z }\right){\fancyscript{f}}_{2}\left(\widehat{t}\right)=\left({I}_{0}{\mu }_{a}{\mathfrak{O}}_{1}\left({e}^{-{e}_{1}\widehat{z}}\right)-{I}_{0}{\mu }_{a}{\mathfrak{O}}_{2}\left({e}^{-{e}_{2}\widehat{z}}\right)\right){\fancyscript{f}}_{2}\left(\widehat{t}\right)$$

Nondimensional initial conditions for the displacement components of skin tissue and temperature are expressed as:

28 $$\theta \left(\widehat{r},\widehat{z},0\right)=\dot{\theta }\left(\widehat{r},\widehat{z},0\right)=\widehat{u}\left(\widehat{r},\widehat{z},0\right)=\dot{\widehat{u}}\left(\widehat{r},\widehat{z},0\right)=\widehat{v}\left(\widehat{r},\widehat{z},0\right)=\dot{\widehat{v}}\left(\widehat{r},\widehat{z},0\right)=0;$$

The nondimensional boundary conditions for $\widehat{u}$ and $\widehat{v}$ along $r$ direction are as follows:

29 $$\underset{\widehat{r}\to \infty }{\mathrm{lim}}\widehat{u}\left(\widehat{r},\widehat{z},\widehat{t}\right)=\widehat{u}\left(0,\widehat{z},\widehat{t}\right)=\underset{\widehat{r}\to \infty }{\mathrm{lim}}\widehat{v}\left(\widehat{r},\widehat{z},\widehat{t}\right)={\left.\frac{\partial \widehat{v}\left(\widehat{r},\widehat{z},\widehat{t}\right)}{\partial \widehat{r}}\right|}_{\widehat{r}=0}=0$$

The nondimensional boundary conditions for $\widehat{u}$ and $\widehat{v}$ along $z$ direction are as follows:

30 $$\underset{\widehat{z}\to \infty }{\mathrm{lim}}\widehat{u}\left(\overline{r },\widehat{z},\overline{t }\right)=\underset{\widehat{z}\to \infty }{\mathrm{lim}}\widehat{v}\left(\overline{r },\widehat{z},\overline{t }\right)=0;$$

31 $$\begin{aligned}&{\left.{\mathfrak{S}}_{rz}\right|}_{\widehat{z}=0}={\left.\left(\frac{\partial \widehat{u}}{\partial \overline{z} }+\frac{\partial \widehat{v}}{\partial \widehat{r}}\right)\right|}_{\widehat{z}=0}=0;\\&{\left.{\mathfrak{S}}_{zz}\right|}_{\widehat{z}=0}={\left.\left\{\left(\frac{\partial \widehat{u}}{\partial \widehat{r}}+\frac{\widehat{u}}{\widehat{r}}\right)+{\mathfrak{M}}_{6}\left(\frac{\partial \widehat{v}}{\partial \widehat{z}}\right)- {\mathfrak{M}}_{7}\theta \left(\widehat{r},\widehat{z},\widehat{t}\right)\right\}\right|}_{\widehat{z}=0}=0\end{aligned}$$

where ${\mathfrak{M}}_{6}=\frac{(\lambda +2\mu )}{\lambda }; {\mathfrak{M}}_{7}=\frac{\mathfrak{B}{\mathcal{T}}_{0}}{\lambda }$. Nondimensional boundary conditions for the temperature along the $r$ and $z$ directions are:

32 $$\underset{\widehat{r}\to \infty }{\mathrm{lim}}\theta \left(\widehat{r},\widehat{z},\widehat{t}\right)=\underset{\widehat{z}\to \infty }{\mathrm{lim}}\theta \left(\widehat{r},\widehat{z},\widehat{t}\right)={\left.\frac{\partial \theta \left(\widehat{r},\widehat{z},\overline{t }\right)}{\partial \overline{r} }\right|}_{\widehat{r}=0}=0;{\left.\frac{\partial \theta \left(\widehat{r},\widehat{z},\widehat{t}\right)}{\partial \widehat{z}}\right|}_{\widehat{z}=0}\cong 0$$

## Numerical method

In this study, the Galerkin-based reduced-order model was utilized to solve the coupled two-dimensional partial differential equations of hyperbolic heat transfer and vibrating bio-tissue equations. Utilizing the Galerkin method, a continuous operator problem, such as a boundary value differential equation is converted to a discrete problem. It is done by applying linear constraints determined by finite sets of basic functions. This method is the equivalent of applying the method of variation of parameters to function space by converting the equation to a weak formulation. It is based on the weak formulation of an equation and limits the possible solutions to a smaller space than the original one, which can be solved more easily. A linear combination of a set of prescribed basis or shape functions is considered for the unknown function. Satisfying the boundary conditions of the problem is dependent on selecting proper shape functions. The accuracy of the solutions depends significantly on the number and type of shape functions.

In this work, complex boundary conditions, especially those related to stresses on the surface of the tissue, appear. To satisfy the complicated boundary conditions, the following approximate solutions are considered for Eqs. (23)–(25).

33 $$\widehat{u}\left(\widehat{r},\widehat{z},\widehat{t}\right)=\sum_{i=1}^{n}\sum_{j=1}^{m}{\mathfrak{Y}}_{i}(\widehat{r}){\mathfrak{F}}_{j}(\widehat{z}){\fancyscript{q}}_{ij}(\widehat{t})+\sum_{i=1}^{n}\sum_{j=1}^{m}{\mathcal{A}}_{i}{\mathfrak{Y}}_{i}(\widehat{r}){{\mathfrak{F}}_{j}^{*}(\widehat{z})\fancyscript{q}}_{ij}\left(\widehat{t}\right)$$

34 $$\widehat{v}\left(\widehat{r},\widehat{z},\widehat{t}\right)=\sum_{p=1}^{a}\sum_{q=1}^{b}{\mathfrak{R}}_{p}(\widehat{r}){\mathfrak{H}}_{q}(\widehat{z}){\fancyscript{p}}_{pq}(\widehat{t})+\sum_{i=1}^{n}\sum_{j=1,}^{m}{\mathcal{B}}_{i}{\mathfrak{R}}_{p}(\widehat{r}){\mathfrak{H}}_{j}^{*}(\widehat{z}){\fancyscript{p}}_{pq}\left(\widehat{t}\right)$$

35 $$\theta \left(\widehat{r},\widehat{z},\widehat{t}\right)=\sum_{k=1}^{u}\sum_{d=1}^{s}{\mathfrak{I}}_{k}(\widehat{r}){\mathfrak{L}}_{d}(\widehat{z}){\fancyscript{s}}_{kd}(\widehat{t})$$

${\mathcal{A}}_{i}$ and ${\mathcal{B}}_{i}$ are time defendant functions and are selected to satisfy the boundary conditions (31). The approximate solutions (33), (34), and (35) are substituted into Eqs. (23), (24), and (25) and the boundary conditions (31) as:

36 $$\begin{aligned}&\sum_{i=1}^{n}\sum_{j=1}^{m}{\mathfrak{Y}}_{i}(\widehat{r})\left({{\mathfrak{F}}_{j}(\widehat{z})+\mathcal{A}}_{i}{\mathfrak{F}}_{j}^{*}(\widehat{z})\right){\ddot{\fancyscript{q}}}_{ij}(\widehat{t})\\&\quad-{\mathfrak{M}}_{1}\sum_{i=1}^{n}\sum_{j=1}^{m}{\mathfrak{Y}}_{i}\left(\widehat{r}\right)\left({\mathfrak{F}}_{j}\left(\widehat{z}\right)+{\mathcal{A}}_{i}{\mathfrak{F}}_{j}^{*}(\widehat{z})\right){\dot{\fancyscript{q}}}_{ij}(\widehat{t})\\&\quad-{\mathfrak{M}}_{2}\sum_{i=1}^{n}\sum_{j=1}^{m}\left({\mathfrak{Y}}_{i}^{\prime\prime}\left(\widehat{r}\right)+\frac{1}{\widehat{r}}{\mathfrak{Y}}_{i}^{\prime}\left(\widehat{r}\right)-\frac{1}{{\widehat{r}}^{2}}{\mathfrak{Y}}_{i}\left(\widehat{r}\right)\right)\left({\mathfrak{F}}_{j}\left(\widehat{z}\right)+{\mathcal{A}}_{i}{\mathfrak{F}}_{j}^{*}\left(\widehat{z}\right)\right)\\&\quad-{\mathfrak{M}}_{3}\left({\mathfrak{Y}}_{i}\left(\widehat{r}\right)\left({\mathfrak{F}}_{j}^{\prime\prime}\left(\widehat{z}\right)+{\mathcal{A}}_{i}{\mathfrak{F}}_{j}^{{*}^{\prime\prime}}\left(\widehat{z}\right)\right)\right){\fancyscript{q}}_{ij}(\widehat{t}) \\&\quad-{\mathfrak{M}}_{4}\sum_{p=1}^{a}\sum_{q=1}^{b}{\mathfrak{R}}_{p}^{\prime}(\widehat{r})\left({\mathfrak{H}}_{q}^{\prime}\left(\widehat{z}\right)+{\mathcal{B}}_{i}{\mathfrak{H}}_{q}^{*\prime}(\widehat{z})\right){\fancyscript{p}}_{pq}(\widehat{t})\\&\quad+{\mathfrak{M}}_{5}\sum_{k=1}^{u}\sum_{d=1}^{s}{\mathfrak{I}}_{k}^{\prime}(\widehat{r}){\mathfrak{L}}_{d}(\widehat{z}){\fancyscript{s}}_{kd}(\widehat{t})={\varepsilon }_{1}\end{aligned}$$

37 $$\begin{aligned} &\sum_{p=1}^{a}\sum_{q=1}^{b}{\mathfrak{R}}_{p}(\widehat{r})\left({{\mathfrak{H}}_{q}(\overline{z })+\mathcal{B}}_{i}{\mathfrak{H}}_{q}^{*}(\widehat{z})\right){\ddot{\fancyscript{p}}}_{pq}(\widehat{t})\\&\quad-{\mathfrak{M}}_{1}\sum_{p=1}^{a}\sum_{q=1}^{b}{\mathfrak{R}}_{p}(\widehat{r})\left({{\mathfrak{H}}_{q}(\overline{z })+\mathcal{B}}_{i}{\mathfrak{H}}_{q}^{*}(\widehat{z})\right){\dot{\fancyscript{p}}}_{pq}(\widehat{t})\\&\quad-{\mathfrak{M}}_{2}\sum_{p=1}^{a}\sum_{q=1}^{b}\left({\mathfrak{R}}_{p}\left(\widehat{r}\right)\left({\mathfrak{H}}_{q}^{\prime\prime}\left(\overline{z }\right){+\mathcal{B}}_{i}{\mathfrak{H}}_{q}^{*\prime\prime}\left(\widehat{z}\right)\right)\right.\\&\left.\quad-{\mathfrak{M}}_{2}\left({\mathfrak{R}}_{p}^{\prime\prime}\left(\widehat{r}\right)+\frac{1}{\widehat{r}}{\mathfrak{R}}_{p}^{\prime}\left(\widehat{r}\right)\right)\left({{\mathfrak{H}}_{q}\left(\widehat{z}\right)+\mathcal{B}}_{i}{\mathfrak{H}}_{q}^{*}(\widehat{z})\right)\right){\fancyscript{p}}_{pq}\left(\widehat{t}\right)\\&\quad-{\mathfrak{M}}_{4}\sum_{i=1}^{n}\sum_{j=1}^{m}\left({\mathfrak{Y}}_{i}^{\prime}\left(\widehat{r}\right)+\frac{1}{\widehat{r}}{\mathfrak{Y}}_{i}\left(\widehat{r}\right)\right)\left({\mathfrak{F}}_{j}^{\prime}\left(\widehat{z}\right)+{\mathfrak{F}}_{j}^{*\prime}\left(\widehat{z}\right)\right){\fancyscript{q}}_{ij}(\widehat{t})\\&\quad+{\mathfrak{M}}_{5}\sum_{k=1}^{u}\sum_{d=1}^{s}{\mathfrak{I}}_{k}(\widehat{r}){\mathfrak{L}}_{d}^{\prime}(\widehat{z}){\fancyscript{s}}_{kd}(\widehat{t})={\varepsilon }_{2}\end{aligned}$$

38 $$\begin{aligned}&\sum_{k=1}^{u}\sum_{d=1}^{s}{{\mathfrak{I}}_{k}(\widehat{r}){\mathfrak{L}}_{d}(\widehat{z})\ddot{\fancyscript{s}}}_{cd}(\widehat{t})\\&\quad+{\mathfrak{N}}_{1}\sum_{i=1}^{n}\sum_{j=1}^{m}\left(\left({\mathfrak{Y}}_{i}^{\prime}\left(\widehat{r}\right)+\frac{1}{\widehat{r}}{\mathfrak{Y}}_{i}\left(\overline{r }\right)\right)\left({\mathfrak{F}}_{j}\left(\widehat{z}\right)+{\mathcal{A}}_{i}{\mathfrak{F}}_{j}^{*}\left(\widehat{z}\right)\right)\right){\ddot{\fancyscript{q}}}_{ij}(\widehat{t})\\&\quad+{\mathfrak{N}}_{1}\sum_{p=1}^{a}\sum_{q=1}^{b}{\mathfrak{R}}_{p}\left(\widehat{r}\right)\left({\mathfrak{H}\Phi }_{q}^{\prime}\left(\widehat{r}\right){+\mathcal{B}}_{i}{\mathfrak{H}}_{q}^{*\prime}\left(\widehat{z}\right)\right){\ddot{\fancyscript{p}}}_{ab}(\widehat{t})\\&\quad+{\mathfrak{N}}_{2}\sum_{k=1}^{u}\sum_{d=1}^{s}{\mathfrak{I}}_{k}(\widehat{r}){\upphi}_{d}(\widehat{z}){\dot{\fancyscript{s}}}_{kd}(\widehat{t})\\&\quad+{\mathfrak{N}}_{3} \sum_{i=1}^{n}\sum_{j=1}^{m}\left(\left({\mathfrak{Y}}_{i}^{\prime}\left(\widehat{r}\right)+\frac{1}{\widehat{r}}{\mathfrak{Y}}_{i}\left(\overline{r }\right)\right)\left({\mathfrak{F}}_{j}(\widehat{z})+{\mathcal{A}}_{i}{\mathfrak{F}}_{j}^{*}(\widehat{z})\right)\right){\dot{\fancyscript{q}}}_{ij}(\widehat{t})\\&\quad+{\mathfrak{N}}_{3}\sum_{p=1}^{a}\sum_{q=1}^{b}{\mathfrak{R}}_{p}\left(\widehat{r}\right)\left({\mathfrak{H}}_{q}^{\prime}\left(\overline{r }\right){+\mathcal{B}}_{i}{\mathfrak{H}}_{q}^{*\prime}\left(\widehat{z}\right)\right){\dot{\fancyscript{p}}}_{pq}(\widehat{t})\\&\quad-\sum_{k=1}^{u}\sum_{d=1}^{s}\left(\left({\mathfrak{N}}_{7}\left({\mathfrak{I}}_{k}^{\prime\prime}\left(\widehat{r}\right)+\frac{1}{\widehat{r}}{\mathfrak{I}}_{k}^{\prime}\left(\overline{r }\right)\right)-\left({\mathfrak{N}}_{4}{\mathfrak{I}}_{k}\left(\overline{r }\right)\right)\right){\mathfrak{L}}_{d}\left(\widehat{z}\right)\right.\\&\quad\left.+{\mathfrak{N}}_{7}{\mathfrak{I}}_{k}\left(\widehat{r}\right){\mathfrak{L}}_{d}^{\prime\prime}\left(\widehat{z}\right)\right){\fancyscript{s}}_{kd}(\widehat{t})-{\mathfrak{N}}_{4}{\theta }_{b}-{\mathfrak{N}}_{5}{\widehat{\mathcal{Q}}}_{\mathcal{L}}\left(\widehat{r},\widehat{z},\widehat{t}\right)-{\mathfrak{N}}_{6}\dot{{\widehat{\mathcal{Q}}}_{\mathcal{L}}}\left(\widehat{r},\widehat{z},\widehat{t}\right)={\varepsilon }_{3}\end{aligned}$$

39 $$\sum_{i=1}^{n}\sum_{j=1}^{m}{\mathfrak{Y}}_{i}\left(\widehat{r}\right)\left({\mathfrak{F}}_{j}^{\prime}\left(0\right)+{\mathcal{A}}_{i}{\mathfrak{F}}_{j}^{*\prime}\left(0\right)\right){\fancyscript{q}}_{ij}\left(\widehat{t}\right)+\sum_{p=1}^{a}\sum_{q=1}^{b}{\mathfrak{R}}_{p}^{\prime}\left(\widehat{r}\right)\left({\mathfrak{H}}_{q}(0)+{\mathcal{B}}_{i}{\mathfrak{H}}_{q}^{*}(0)\right){\fancyscript{p}}_{pq}(\widehat{t})={\varepsilon }_{4}$$

40 $$\begin{aligned}&\sum_{i=1}^{n}\sum_{j=1}^{m}\left(\left({\mathfrak{Y}}_{i}^{\prime}\left(\widehat{r}\right)+\frac{1}{\overline{r}}{\mathfrak{Y} }_{i}\left(\widehat{r}\right)\right)\left({\mathfrak{F}}_{j}\left(0\right)+{\mathcal{A}}_{i}{\mathfrak{F}}_{j}^{*}\right)\right){\fancyscript{q}}_{ij}(\widehat{t})\\&\quad+{\mathfrak{M}}_{6}\sum_{p=1}^{a}\sum_{q=1}^{b}{\mathfrak{R}}_{p}\left(\widehat{r}\right)\left({\mathfrak{H}}_{q}^{\prime}(0)+{\mathcal{B}}_{i}{\mathfrak{H}}_{q}^{*\prime}(0)\right){\fancyscript{p}}_{pq}(\widehat{t})\\&\quad-{\mathfrak{M}}_{7}\sum_{k=1}^{u}\sum_{d=1}^{s}{\mathfrak{I}}_{k}(\widehat{r}){\mathfrak{L}}_{d}(0){\fancyscript{s}}_{kd}(\widehat{t})={\varepsilon }_{5}\end{aligned}$$

Applying the Galerkin integrals as:

41 $${\int }_{0}^{\infty }{\int }_{0}^{\infty }{\varepsilon }_{1}{\mathfrak{Y}}_{e}\left(\widehat{r}\right){\mathfrak{F}}_{f}\left(\widehat{z}\right)d\widehat{r}d\widehat{z}=0 \quad e=\mathrm{1,2},\dots .n, f=\mathrm{1,2},\dots .m$$

42 $${\int }_{0}^{\infty }{\int }_{0}^{\infty }{\varepsilon }_{2}{\mathfrak{R}}_{g}(\overline{r }){\mathfrak{H}}_{h}(\overline{z })d\widehat{r}d\widehat{z}=0 \quad g=\mathrm{1,2},\dots .p, h=\mathrm{1,2},\dots .q$$

43 $${\int }_{0}^{\infty }{\int }_{0}^{\infty }{\varepsilon }_{3}{\mathfrak{I}}_{c}(\widehat{r}){\mathfrak{L}}_{l}(\overline{z })d\widehat{r}d\widehat{z}=0 \quad c=\mathrm{1,2},\dots .u, l=\mathrm{1,2},\dots .s$$

44 $${\int }_{0}^{\infty }{\varepsilon }_{4}{\mathfrak{Y}}_{e}(\widehat{r})d\widehat{r}=0 \quad e=\mathrm{1,2},3,\dots n$$

45 $${\int }_{0}^{\infty }{\varepsilon }_{5}{\mathfrak{R}}_{g}(\widehat{r})d\widehat{r}=0 g=\mathrm{1,2},3,\dots .a$$

The following equations are obtained:

46 $$\begin{aligned}&\sum_{i=1}^{n}\sum_{j=1}^{m}{M}_{ei}^{\left(1\right)}\left({M}_{fj}^{\left(2\right)}+{\mathcal{A}}_{i}{M}_{fj}^{\left(3\right)}\right){\ddot{\fancyscript{q}}}_{ij}(\widehat{t})-\sum_{i=1}^{n}\sum_{j=1}^{m}{C}_{ei}^{\left(1\right)}\left({C}_{fj}^{\left(2\right)}+{\mathcal{A}}_{i}{C}_{fj}^{\left(3\right)}\right){\ddot{\fancyscript{q}}}_{ij}(\widehat{t})\\&\quad-\sum_{i=1}^{n}\sum_{j=1}^{m}\left({K}_{ei}^{\left(1\right)}\left({K}_{fj}^{\left(2\right)}+{\mathcal{A}}_{i}{K}_{fj}^{\left(3\right)}\right)+{K}_{ei}^{\left(4\right)}\left({K}_{fj}^{\left(5\right)}+{\mathcal{A}}_{i}{K}_{fj}^{\left(6\right)}\right)\right){\fancyscript{q}}_{ij}(\widehat{t})\\&\quad-\sum_{p=1}^{a}\sum_{q=1}^{b}{E}_{ep}^{\left(1\right)}\left({{E}_{fq}^{\left(2\right)}+{\mathcal{B}}_{i}E}_{fq}^{\left(3\right)}\right){\fancyscript{p}}_{pq}(\widehat{t})+\sum_{k=1}^{u}\sum_{d=1}^{s}{F}_{ek}^{\left(1\right)}{F}_{fd}^{\left(2\right)}{\fancyscript{s}}_{kd}(\widehat{t})=0 \end{aligned}$$

47 $$\begin{aligned}&\sum_{p=1}^{a}\sum_{q=1}^{b}{M}_{gp}^{(4)}\left({M}_{hq}^{(5)}+{\mathcal{B}}_{i}{M}_{hq}^{(6)}\right){\ddot{\fancyscript{p}}}_{pq}(\widehat{t})-\sum_{p=1}^{a}\sum_{q=1}^{b}{C}_{gp}^{(4)}\left({C}_{hq}^{(5)}+{\mathcal{B}}_{i}{C}_{hq}^{(6)}\right){\ddot{\fancyscript{p}}}_{pq}(\widehat{t})\\&\quad-\sum_{p=1}^{a}\sum_{q=1}^{b}\left({E}_{gp}^{\left(4\right)}\left({E}_{hq}^{\left(5\right)}+{\mathcal{B}}_{i}{E}_{hq}^{\left(6\right)}\right)+{E}_{gp}^{\left(7\right)}\left({E}_{gp}^{\left(8\right)}+{\mathcal{B}}_{i}{E}_{gp}^{\left(9\right)}\right)\right){\fancyscript{p}}_{pq}\left(\widehat{t}\right)\\&\quad-\sum_{i=1}^{n}\sum_{j=1}^{m}\left({K}_{gi}^{\left(7\right)}\left({K}_{hj}^{\left(8\right)}+{\mathcal{A}}_{i}{K}_{hj}^{\left(9\right)}\right)\right){\fancyscript{q}}_{ij}\left(\widehat{t}\right)\\&\quad+\sum_{k=1}^{u}\sum_{d=1}^{s}({F}_{gk}^{\left(3\right)}{F}_{hd}^{\left(4\right)}) {\fancyscript{s}}_{cd}(\widehat{t})=0 \end{aligned}$$

48 $$\begin{aligned}&\sum_{k=1}^{u}\sum_{d=1}^{s}\left({M}_{ck}^{(7)}{M}_{ld}^{(8)}\right){\ddot{\fancyscript{s}}}_{kd}(\widehat{t})\\&\quad+\sum_{i=1}^{n}\sum_{j=1}^{m}{M}_{ci}^{(9)}({M}_{lj}^{(10)}+{\mathcal{A}}_{i}{M}_{lj}^{(11)}){\ddot{\fancyscript{q}}}_{ij}\left(\widehat{t}\right)\\&\quad+\sum_{p=1}^{a}\sum_{q=1}^{b}{M}_{cp}^{\left(12\right)}\left({M}_{lq}^{\left(13\right)}+{\mathcal{B}}_{i}{M}_{lq}^{\left(14\right)}\right){\ddot{\fancyscript{p}}}_{pq}(\widehat{t})\\&\quad+\sum_{k=1}^{u}\sum_{d=1}^{s}{(C}_{ck}^{\left(7\right)}{C}_{ld}^{\left(8\right)}{\dot{)\fancyscript{s}}}_{kd}\left(\widehat{t}\right)\\&\quad+\sum_{i=1}^{n}\sum_{j=1}^{m}{C}_{ci}^{\left(9\right)}\left({C}_{lj}^{\left(10\right)}+{\mathcal{A}}_{i}{C}_{lj}^{\left(11\right)}\right){\dot{\fancyscript{q}}}_{ij}\left(\widehat{t}\right)\\&\quad+\sum_{p=1}^{a}\sum_{q=1}^{b}{C}_{cp}^{\left(12\right)}\left({C}_{lq}^{\left(13\right)}+{\mathcal{B}}_{i}{C}_{lq}^{\left(14\right)}\right){\dot{\fancyscript{p}}}_{pq}\left(\widehat{t}\right)\\&\quad-\sum_{k=1}^{u}\sum_{d=1}^{s}\left({F}_{ck}^{\left(5\right)}{F}_{ld}^{\left(6\right)}+{F}_{ck}^{\left(7\right)}{F}_{ld}^{\left(8\right)}\right){\fancyscript{s}}_{kd}(\widehat{t})-{P}_{cl}^{\left(1\right)} -{P}_{cl}^{\left(2\right)}(\widehat{t})-{P}_{cl}^{\left(3\right)}(\widehat{t})=0 \end{aligned}$$

49 $$\sum_{i=1}^{n}\sum_{j=1}^{m}{K}_{ei}^{(10)}\left({K}_{j}^{(11)}+{\mathcal{A}}_{i}{K}_{j}^{(12)}\right)){\fancyscript{q}}_{ij}\left(\widehat{t}\right)+\sum_{p=1}^{a}\sum_{q=1}^{b}{{E}_{ep}^{\left(10\right)}\left({E}_{q}^{\left(11\right)}+{\mathcal{B}}_{i}{E}_{q}^{\left(12\right)}\right)\fancyscript{p}}_{pq}\left(\widehat{t}\right)=0 $$

50 $$\begin{aligned}&\sum_{i=1}^{n}\sum_{j=1}^{m}{K}_{gi}^{(13)}\left({K}_{j}^{(14)}+{\mathcal{A}}_{i}{K}_{j}^{(15)}\right){\fancyscript{q}}_{ij}\left(\widehat{t}\right)\\&\quad+\sum_{p=1}^{a}\sum_{q=1}^{b}{E}_{gp}^{\left(13\right)}\left({E}_{q}^{\left(14\right)}+{\mathcal{B}}_{i}{E}_{q}^{\left(15\right)}\right){E}_{q}^{\left(15\right)}{\fancyscript{p}}_{pq}(\widehat{t})\\&\quad-\sum_{k=1}^{u}\sum_{d=1}^{s}{F}_{gk}^{\left(9\right)}{F}_{d}^{\left(10\right)}{\fancyscript{s}}_{kd}(\widehat{t})=0 \end{aligned}$$

with the coefficients listed in “Appendix A”.

The following shape functions are considered for solving the equations:

$${\mathfrak{Y}}_{i}\left(\widehat{r}\right)={J}_{I}\left(i\widehat{r}\right);{\mathfrak{R}}_{p}\left(\widehat{r}\right)=\mathrm{cos}\left(p\pi \widehat{r}\right).\mathrm{exp}\left(-p{\widehat{r}}^{2}\right);{\mathfrak{I}}_{k}\left(\widehat{r}\right)={e}^{-k{\widehat{r}}^{2}};{\mathfrak{F}}_{j}\left(\widehat{z}\right)={e}^{-j{\widehat{z}}^{2}}$$

51 $${\mathfrak{F}}_{j}^{*}\left(\widehat{z}\right)={\widehat{z}e}^{-j\widehat{z}};{\mathfrak{H}}_{q}\left(\widehat{z}\right)={e}^{-q{\widehat{z}}^{2}};{\mathfrak{H}}_{q}^{*}\left(\widehat{z}\right)={\widehat{z}e}^{-q\widehat{z}};{\mathfrak{L}}_{d}\left(\widehat{z}\right)={e}^{-d{\widehat{z}}^{2}}$$

where ${J}_{I}$ is the Bessel function of the first kind.

Considering $n=m=a=b=u=s=1$, in Eqs. (46–50), the parameters $\mathcal{A}$ and $\mathcal{B}$ can be obtained as:

52 $${\mathcal{A}}_{1}={\mathfrak{d}}_{1}\frac{{\fancyscript{p}}_{11}}{{\fancyscript{q}}_{11}};{\mathcal{B}}_{1}={\mathfrak{d}}_{2}\frac{{\fancyscript{q}}_{11}}{{\fancyscript{p}}_{11}}+{\mathfrak{d}}_{3}\frac{{\fancyscript{s}}_{11}}{{\fancyscript{p}}_{11}}$$

in which ${\mathfrak{d}}_{1}$, ${\mathfrak{d}}_{2}$ and ${\mathfrak{d}}_{3}$ are defined as:

53 $${\mathfrak{d}}_{1}=-\frac{{E}_{11}^{(10)}{E}_{11}^{(11)}}{{K}_{11}^{(10)}{K}_{11}^{(12)}}; {\mathfrak{d}}_{2}=-\frac{{K}_{11}^{(13)}{K}_{11}^{(14)}}{{E}_{11}^{(13)}{E}_{11}^{(15)}};{\mathfrak{d}}_{3}=\frac{{F}_{11}^{(9)}{F}_{11}^{(10)}}{{E}_{11}^{(13)}{E}_{11}^{(15)}}$$

Substituting the parameters $\mathcal{A}$ and $\mathcal{B}$ into Eqs. (46)–(48), they simplify to:

54 $${\mathcal{M}}_{11}^{(1)}{\ddot{\fancyscript{q}}}_{11}+{\mathcal{M}}_{11}^{(2)}{\ddot{\fancyscript{p}}}_{11}+{\mathcal{C}}_{11}^{(1)}{\dot{\fancyscript{q}}}_{11}+{\mathcal{C}}_{11}^{(2)}{\dot{\fancyscript{p}}}_{11}+{\mathcal{K}}_{11}^{(1)}{\fancyscript{q}}_{11}+{\mathcal{K}}_{11}^{(2)}{\fancyscript{p}}_{11}+{\mathcal{K}}_{11}^{(3)}{\fancyscript{s}}_{11}=0$$

55 $${\mathcal{M}}_{11}^{\left(3\right)}{\ddot{\fancyscript{p}}}_{11}+{\mathcal{M}}_{11}^{\left(4\right)}{\ddot{\fancyscript{q}}}_{11}+{\mathcal{M}}^{\left(5\right)}{\ddot{\fancyscript{s}}}_{11}+{\mathcal{C}}_{11}^{\left(3\right)}{\dot{\fancyscript{p}}}_{11}+{\mathcal{C}}_{11}^{\left(4\right)}{\dot{\fancyscript{q}}}_{11}+{\mathcal{C}}^{\left(5\right)}{\dot{\fancyscript{s}}}_{11}+{\mathcal{K}}_{11}^{\left(4\right)}{\fancyscript{p}}_{11}+{\mathcal{K}}_{11}^{(5)}{\fancyscript{q}}_{11}+{\mathcal{K}}_{11}^{(6)}{\fancyscript{s}}_{11}=0$$

56 $${\mathcal{M}}_{11}^{(6)}{\ddot{\fancyscript{s}}}_{11}+{\mathcal{M}}_{11}^{(7)}{\ddot{\fancyscript{q}}}_{11}+{\mathcal{M}}_{11}^{(8)}{\ddot{\fancyscript{p}}}_{11}+{\mathcal{C}}_{11}^{(6)}{\dot{ \xi }}_{11}+{\mathcal{C}}_{11}^{(7)}{\dot{\fancyscript{q}}}_{11}+{\mathcal{C}}_{11}^{(8)}{\dot{\fancyscript{p}}}_{11}+{\mathcal{K}}^{(7)}{\fancyscript{s}}_{11}-{P}_{11}^{(1)}-{P}_{11}^{(2)}-{P}_{11}^{\left(3\right)}=0$$

with the coefficients listed in “Appendix B”.

Equations (54–56) are solved simultaneously to obtain the transient temperature and vibration response of the skin tissue.

## Numerical results

For numerical calculation, values of the heat and physical properties of the skin tissue, blood, and the parameters of the laser heat source are listed in Table 1.

**Table 1:** Properties of the skin tissue and the laser heat source^25,45^.

Density of tissue $\left(\rho \right)$	1000 $\left(\mathrm{kg}/{\mathrm{m}}^{3}\right)$
Density of blood $\left({\rho }_{b}\right)$	1060 $\left(\mathrm{kg}/{\mathrm{m}}^{3}\right)$
Specific heat of the tissue $\left(c\right)$	4187 $\left(\mathrm{J}/\mathrm{kg}.\mathrm{K}\right)$
Specific heat of blood $\left({c}_{b}\right)$	3860 $\left(\mathrm{J}/\mathrm{kg}.\mathrm{K}\right)$
Heat conductivity of tissue $\left(k\right)$	0.628 $\left(\mathrm{W}/\mathrm{m}.\mathrm{K}\right)$
Blood perfusion $\left({G}_{b}\right)$	1.87E−3 $\left(1/\mathrm{s}\right)$
Relaxation time $\left(\tau \right)$	3 $\left(\mathrm{s}\right)$
Elasticity modulus of tissue $\left(E\right)$	100 $\left(\mathrm{MPa}\right)$
Thermal expansion $\left({\alpha }_{T}\right)$	1E-4 $\left(1/\mathrm{K}\right)$
Poisson’s ratio $\left(\upsilon \right)$	0.4
Laser intensity $\left({I}_{0}\right)$	3E+5 $\left(\mathrm{W}/{\mathrm{m}}^{2}\right)$
Diffuse reflectance $\left({R}_{d}\right)$	0.05
Reference temperature $\left({T}_{0}\right)$	37 °C

**Figure 2:**
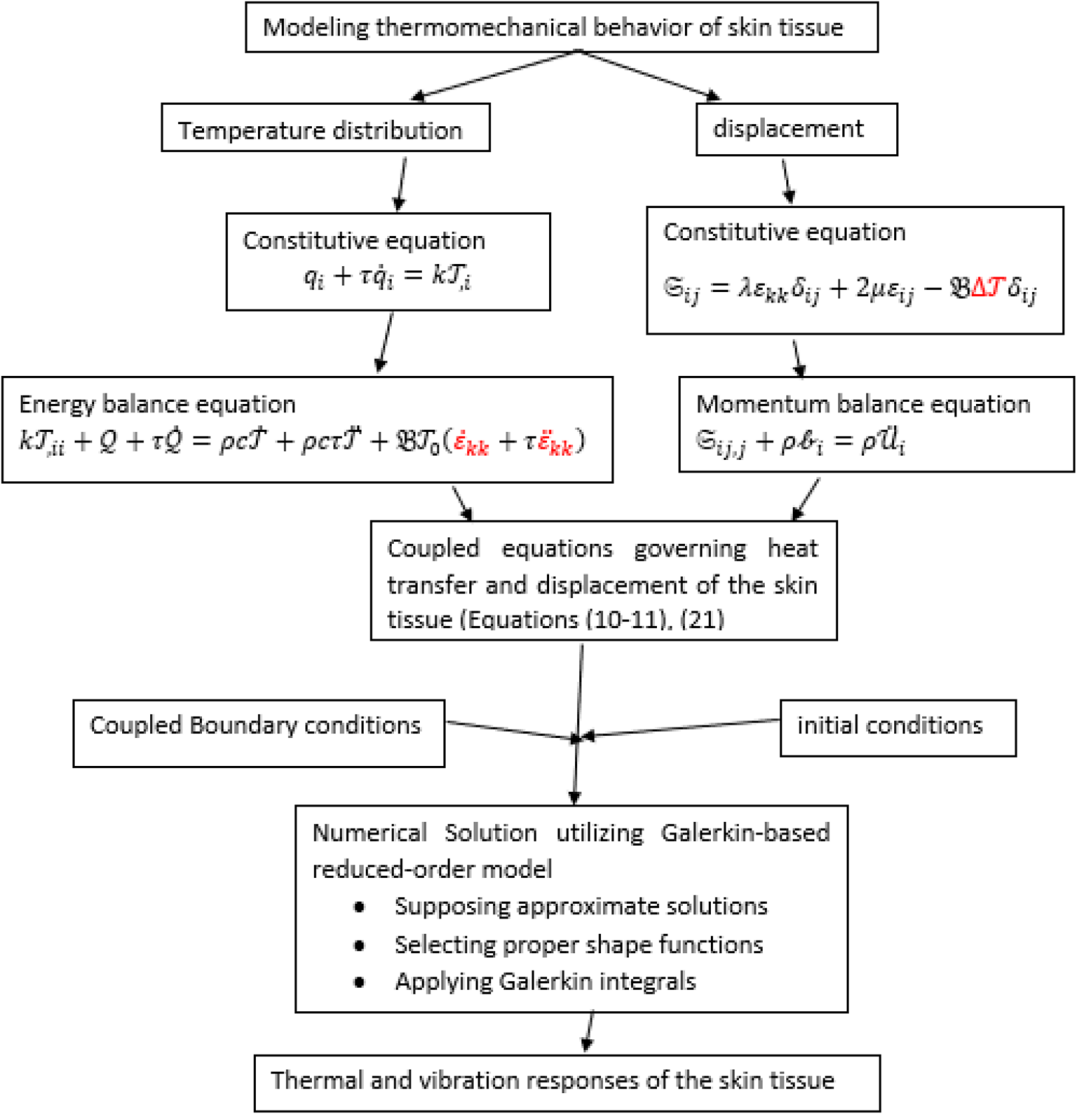
Flow chart of the modeling and numerical method of the problem.

For the modeling and its solution to be more understandable, a clear flow chart of the modeling and the numerical method is presented in Fig. 2. The coupling terms in equations are shown in red font in the chart.

In this section, the transient thermomechanical response of skin tissue subjected to different types of heating sources is investigated.

### Single-pulse heating source

**Figure 3:**
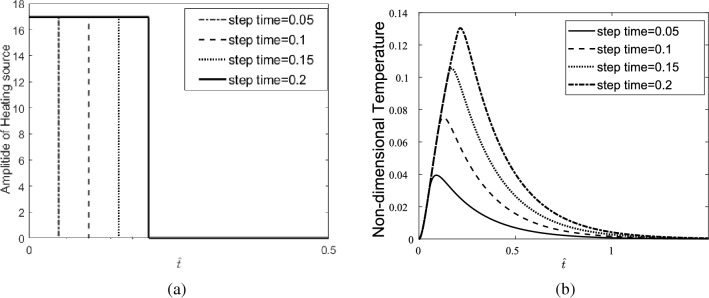
(**a**) Single-pulse heating source, (**b**) temperature response of the skin tissue to a single-pulse heat source with different step times.

Considering the single pulse heating source shown in Fig. 3a, the temperature response of the skin tissue is presented and shown in Fig. 3b.

**Figure 4:**
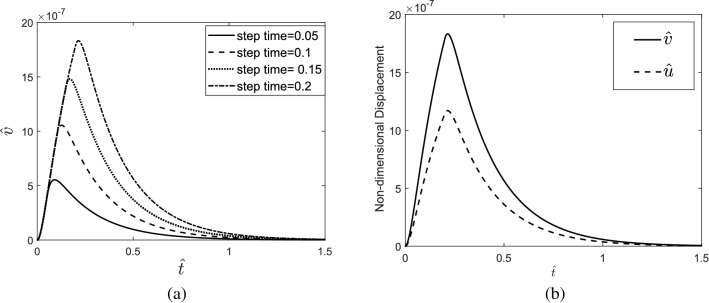
(**a**) Displacement response of the skin tissue in the z-direction to a single-pulse heat source with different step times, (**b**) comparison of the displacement response of the skin tissue in the depth direction with its response in the radial direction.

The results indicate that in a laser single pulse heating source with high step time, the tissue can reach higher temperatures than a heating source with low step time. It is also observed that the step time does not affect the gradient of temperature in the heating process but increases the temperature gradient in the cooling process. The displacement response of tissue in the depth direction (z-direction) is shown in Fig. 4a, and in Fig. 4b, it is compared to the displacement response of tissue in the radial direction (r-direction).

**Figure 5:**
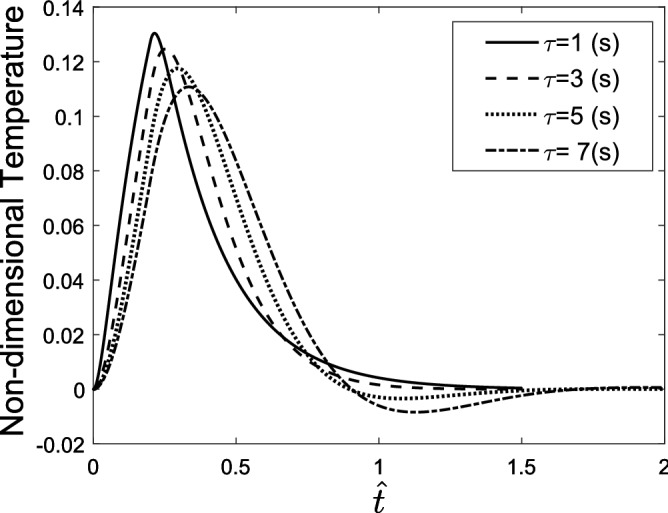
Temperature response of the skin tissue considering different relaxation time values.

A higher step time increases the maximum displacement of the tissue and reaches the maximum temperature. It also increases the displacement gradient of tissue in the cooling process, while it does not affect the gradient of displacement in the heating process. Figure 5 shows the temperature of the skin tissue versus time considering different values of relaxation time. It was mentioned that in the generalized Fourier law, an inertial term is considered for the heat transfer phenomenon, which is concerned with the relaxation time. An increase in the relaxation time leads to an increase in the inertial effects and therefore causes the reached maximum temperature to decrease.

### Repetitive pulse heating source

**Figure 6:**
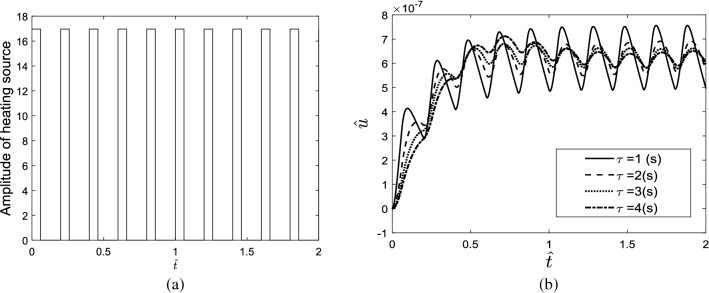
(**a**) Repetitive pulse heating source, (**b**) displacement response of the skin tissue considering different relaxation times.

Considering the repetitive pulse heating source shown in Fig. 6a, the displacement response of the skin tissue is shown in Fig. 6b. The results indicate that increasing relaxation time decreases the temperature gradient at the beginning of the heating process. In other words, relaxation time decreases the first maximum overshoot and increases the pick time in the response of the skin tissue. Relaxation time also reduces the amplitude of temperature vibrations of the skin in the whole heating process. In this work, the heat conduction phenomenon in the skin tissue is modeled based on the non-Furies conduction equation which is attributed to the Cattaneo–Vernotte model. In the presented model, $\tau$ is the relaxation time. It is a function of the material. It is defined as the time necessary for storage of the thermal energy required for the propagative transfer to an element in thermal contact. In other words, the term $\tau \frac{\partial q}{\partial t}$ in the non-Fourie’s heat conduction equation behaves as an inertial term in the conduction phenomenon which restricts the infinite propagation speed in the bio tissue. So, by increasing the relaxation time, the effect of the thermal inertia term increases. Therefore, the maximum temperature that the skin can be reached during thermal radiation decreases. Inertia term also creates a delay in the thermal and vibrating response of the tissue that leads to the increment of peak time in the time-domain thermomechanical response of the tissue.

**Figure 7:**
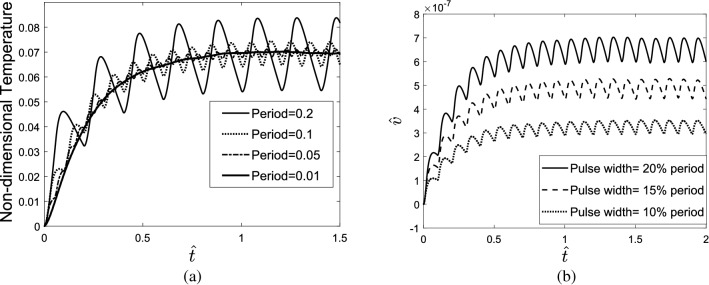
(**a**) The effect of the period, (**b**) the effect of the width of the repetitive pulses on the thermomechanical response of the tissue.

The effect of the period and width of the repetitive pulses on the thermomechanical response of the tissue was investigated. The indicated results in Fig. 7a,b show that when the period of the pulses reduces, the amplitude of temperature vibrations decreases; therefore, the temperature response of tissue will be similar to the response subjected to the step heating source. Moreover, by increasing the width of laser pulses, the tissue can reach high temperatures during the heating process.

### Repeating sequence stair

**Figure 8:**
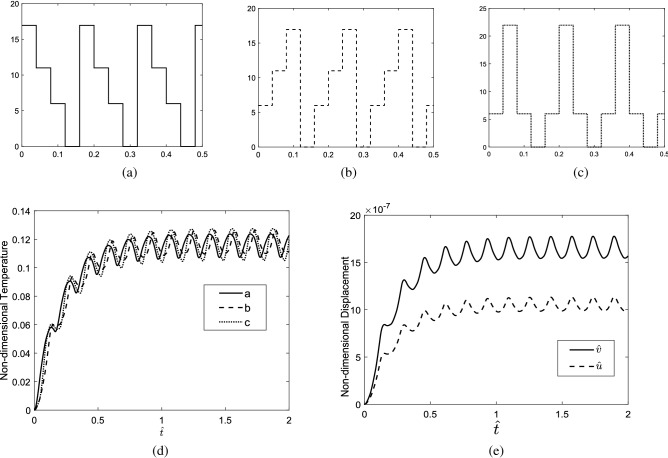
(**a**–**c**) Different types of repeating sequence stairs, (**d**) non-dimensional temperature of tissue considering different types of repeating sequence stairs heating sources, (**e**) time-domain displacement response of tissue in radial and depth direction.

Different types of repeating sequence stair heating sources are shown in Fig. 8a,b,c in which the equal energy is delivered to the tissue in all cases. In Fig. 8a, the sequence stairs are applied in the cooling stage, while in Fig. 8b, the sequence stairs are applied in the heating stage, and in Fig. 8c, symmetric sequence stairs are applied in the heating and cooling stages of the skin heating. The non-dimensional temperature of the skin tissue versus time is shown in Fig. 8d). When sequence stairs are applied in the heating stage, the tissue can experience a higher maximum temperature than when they are applied in the cooling stage. In other words, heating the skin gradually by sequence stairs and cooling it suddenly, tissue can experience higher temperature rise compared to the case when it is heated suddenly and cooled gradually. When the tissue encounters thermal shock whether, in the heating or cooling process (cases (a), and (b)), it delivers or losses the energy suddenly. Therefore, the high percentage of the delivered energy is converted to mechanical energy which leads to the vibration of the tissue rather than the increment of its temperature. However, in case (c) where the tissue is heated and cooled gradually via sequence stairs, most of the delivered energy is used to increase the internal energy of tissue rather than its displacement. Therefore, the maximum temperature, the tissue can be reached via both heating and cooling with sequence stairs (case (c)) is high compared with cases (a), and (b). In Fig. 8e) the time-domain displacement response of the tissue along radial and depth direction is presented. The displacement of tissue in the depth direction is more considerable than the radial direction.

### Step heating source

**Figure 9:**
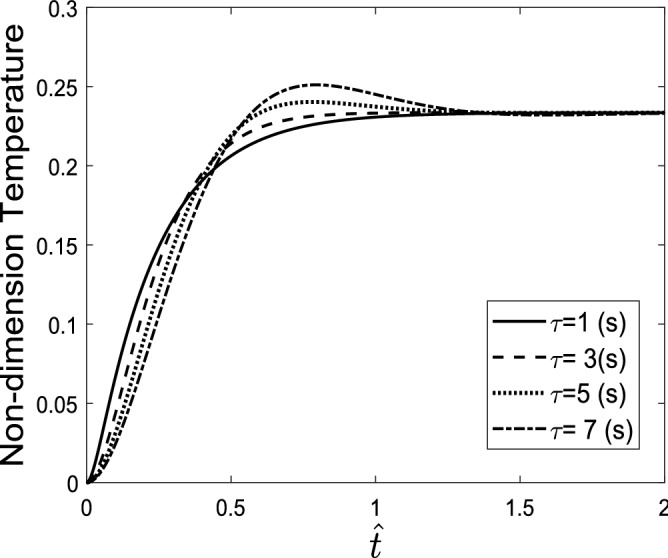
Temperature response of the skin tissue considering the step heating source.

**Figure 10:**
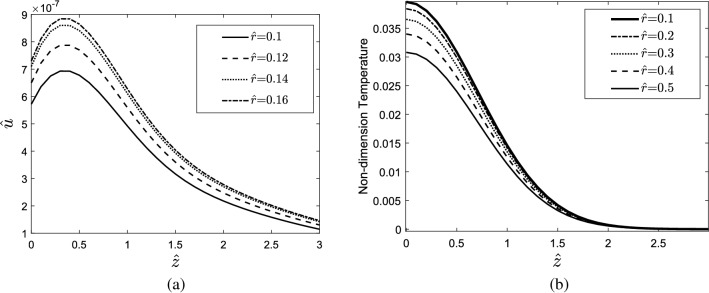
(**a**) Nondimensional displacement of tissue through the z-direction versus $\widehat{z}$ for different values of $\widehat{r}$ and (**b**) temperature distribution through the z-direction for different values of $\widehat{r}$.

Figure 9 shows the temperature response of the skin tissue, considering a step heating source. At very low relaxation times, the skin tissue does not experience any overshoot in the thermal response. Increasing the relaxation time may lead the tissue to encounter an overshoot during its transient response. The nondimensional displacement of tissue along the z-direction at $\widehat{t}=0.1$ for different values of $\widehat{r}$ is shown in Fig. 10a,b. The results indicate that by moving away from the center of the tissue where the pulse heating source is applied, the displacement of tissue in the r- and z-directions decreases and eventually vanishes.

### Repetitive ramp heating source

**Figure 11:**
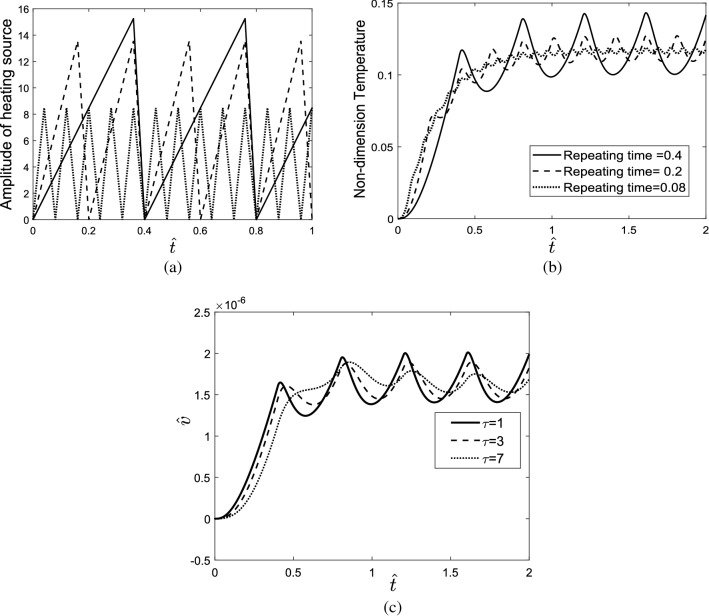
(**a**) Repetitive ramp heating source, (**b**) temperature response of the skin tissue considering different repeating times, (**c**) time-domain response of tissue along depth direction for different values of relaxation time.

Figure 11a shows the respective ramp heating source with different repeating times, and Fig. 11b) represents the transient response of the tissue. Reducing the repeating time decreases the vibrating amplitude of temperature, therefore causing the skin to experience a low-temperature rise. In Fig. 11c the vibration response of the tissue considering different relaxation times is presented. In lower relaxation time, the tissue encounters with higher overshoot in the time-domain response of it.

### Harmonic heating source

**Figure 12:**
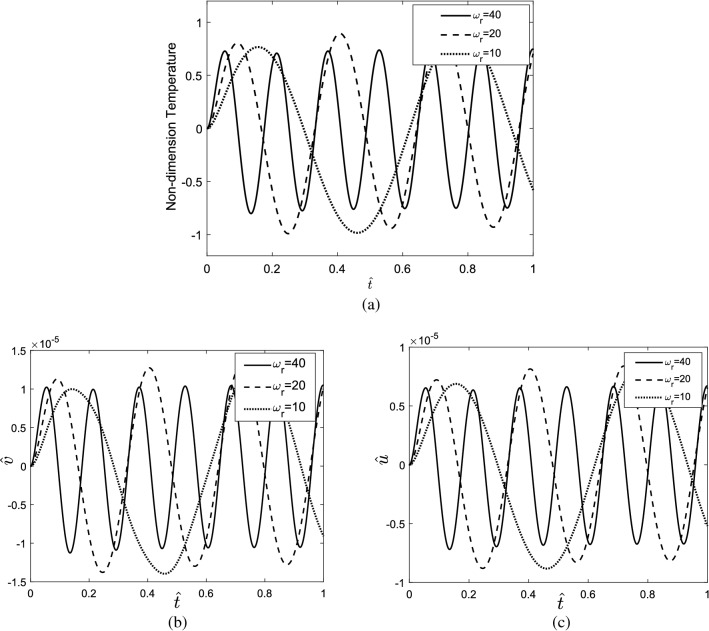
(**a**) Temperature response of the skin tissue, (**b**) displacement of the tissue along radial direction, (**c**) displacement of the tissue along the depth direction, to different frequency ratios.

The transient thermal response of the skin tissue is studied supposing a harmonic heating source. Figure 12a shows the temperature response of tissue to the harmonic heating source considering different value frequency ratios $\left({\omega }_{r}\right)$. The frequency ratio is defined as the ratio of the excitation frequency to the characteristic frequency of the tissue ($\frac{\alpha }{{\fancyscript{h}}^{2}}$). The frequency ratio displaces the time at which the tissue reaches the maximum temperature. Figure 12b,c show the transient response of tissue along with depth and z-direction.

### Validation of the results

**Figure 13:**
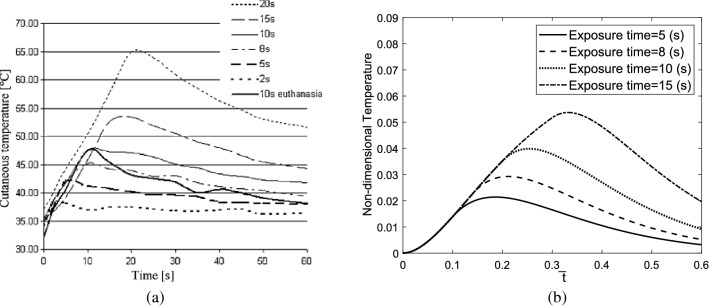
(**a**) The obtained experimental study reported by Mseux et al., considering laser power of $122\frac{kW}{{m}^{2}}$^54^, (**b**) numerical results obtained in this work giving a single laser pulse.

The results obtained in this work are based on the human skin tissue with the properties listed in Table 1. The blood properties and laser parameters based on which the results are calculated are also listed in Table 1. In Some experimental studies, it has been shown that there are great similarities between human and pig skins, especially in the vascular organization. An experimental study was conducted on laser heating of pig skins considering a single pulse with various exposure duration times and laser powers^54^. The reported experimental data are shown in Fig. 13a). Figure 13b) indicates the numerical results calculated in this work considering a single laser pulse with a laser power of $122\frac{kW}{{m}^{2}}$. Neglecting the effect of the thermoelastic coupling term, the maximum temperature the skin reaches several exposure durations is listed in Table 2. A good agreement is observed between the results of the presented numerical solution and those obtained from the experimental study.

**Table 2:** The maximum temperature of the skin tissue obtained in this study for different exposure times.

Exposure time (step time)	5(s)	8(3)	10(s)	15(s)
Maximum temperature of the skin tissue (°C)	43.8	45.6	49.4	53.1

The results of the presented coupled thermoelastic model are compared with the results obtained based on the finite element method^55^. Therefore, by considering the initial and boundary conditions, the material properties are the same as those used in Ref.^55^, the maximum temperature of the skin tissue in two different points in the skin tissue is compared with the obtained values in Ref.^55^. The results are presented in Table 3. The obtained maximum temperature of the tissue shows agreement with the results obtained based on FEM. It shows that the present numerical method is effective and accurate.

**Table 3:** Comparison of the maximum temperature of the skin tissue obtained in this study with the obtained values in Ref^55^.

Position of point in the skin tissue	$r=0;z=0.157$	$r=0.5;z=0.157$
Maximum temperature of the skin tissue (°C) in this study	55.3	44.2
Maximum temperature of the skin tissue (°C) in Ref.^55^	56.2	45.7

## Conclusion

In the present study, the thermomechanical behavior of the skin tissue exposed to different types of heating sources was presented. 2-D coupled differential equations of the hyperbolic heat transfer and 2-D dynamic displacement of tissue were extracted, considering the thermoelastic coupling term. The mixed accompanying boundary conditions (displacement and force) governing the displacement equation that has made the solution of the equations so complicated were imposed using a heuristic method. A Galerkin-based reduced-order model was utilized to solve the coupled equations with mixed boundary conditions. The transient temperature and displacement response of the tissue were analyzed under different types of thermal heating loading. It was shown that when a single pulse heating source with a high step time is applied on the skin, the tissue experiences a higher temperature rise. No changes in the temperature gradient were observed in the heating stage of the process. However, the temperature gradient in the cooling process was considerable. In the case of repetitive pulses, a decrease in the period of the pulses led to a reduction in the amplitude of the temperature vibrations. It was observed that when a sequence stair is applied in the heating stage, the tissue can reach a higher maximum temperature compared with the case when it is applied in the cooling stage. Increasing the relaxation time in the step thermal loading caused the tissue to experience an overshoot during its transient response. Reducing the repeating time in the ramp-type heating source decreased the amplitude of the temperature vibration.

## Appendix A

$${M}_{ei}^{(1)}={\int }_{0}^{\infty }{\mathfrak{Y}}_{e}\left(\widehat{r}\right){\mathfrak{Y}}_{i}\left(\widehat{r}\right)d\widehat{r};{M}_{fj}^{(2)}={\int }_{0}^{\infty }{\mathfrak{F}}_{f}\left(\widehat{z}\right){\mathfrak{F}}_{j}\left(\widehat{z}\right)d\widehat{z};{M}_{fj}^{(3)}={\int }_{0}^{\infty }{\mathfrak{F}}_{f}(\widehat{z}){\mathfrak{F}}_{j}^{*}(\widehat{z})d\widehat{z};$$

$${C}_{ei}^{(1)}={\mathfrak{M}}_{1}{\int }_{0}^{\infty }{\mathfrak{Y}}_{e}\left(\widehat{r}\right){\mathfrak{Y}}_{i}\left(\widehat{r}\right)d\widehat{r};{C}_{fj}^{(2)}={\int }_{0}^{\infty }{\mathfrak{F}}_{f}\left(\widehat{z}\right){\mathfrak{F}}_{j}\left(\widehat{z}\right)d\widehat{z};{C}_{fj}^{(3)}={\int }_{0}^{\infty }{\mathfrak{F}}_{f}(\widehat{z}){\mathfrak{F}}_{j}^{*}(\widehat{z})d\widehat{z};$$

$${K}_{ei}^{(1)}={\mathfrak{M}}_{2}{\int }_{0}^{\infty }{\mathfrak{Y}}_{e}\left(\widehat{r}\right)\left({\mathfrak{Y}}_{i}^{\prime\prime}\left(\widehat{r}\right)+\frac{1}{\widehat{r}}{\mathfrak{Y}}_{i}^{\prime}\left(\widehat{r}\right)-\frac{1}{{\widehat{r}}^{2}}{\mathfrak{Y}}_{i}\left(\widehat{r}\right)\right)d\widehat{r};{K}_{fj}^{(2)}={\int }_{0}^{\infty }{\mathfrak{F}}_{f}(\widehat{z}){\mathfrak{F}}_{j}(\widehat{z})d\widehat{z};$$

$${K}_{fj}^{(3)}={\int }_{0}^{\infty }{\mathfrak{F}}_{f}(\widehat{z}){\mathfrak{F}}_{j}^{*}(\widehat{z})d\widehat{z};{K}_{ei}^{(4)}={\mathfrak{W}}_{3}{\int }_{0}^{\infty }{\mathfrak{Y}}_{e}\left(\widehat{r}\right){\mathfrak{Y}}_{i}\left(\widehat{r}\right)d\widehat{r};{K}_{fj}^{(5)}={\int }_{0}^{\infty }{\mathfrak{F}}_{f}(\widehat{z}){\mathfrak{F}}_{j}^{\prime\prime}(\widehat{z})d\widehat{z}$$

$${K}_{fj}^{(6)}={\int }_{0}^{\infty }{\mathfrak{F}}_{f}(\widehat{z}){\mathfrak{F}}_{j}^{*\prime\prime}(\widehat{z})d\widehat{z};{E}_{ep}^{(1)}={\mathfrak{W}}_{4}{\int }_{0}^{\infty }{\mathfrak{Y}}_{e}(\widehat{r}){\mathfrak{R}}_{p}^{\prime}\left(\widehat{r}\right)d\widehat{r};{E}_{fq}^{(2)}={\int }_{0}^{\infty }{\mathfrak{F}}_{f}(\widehat{z}){\mathfrak{H}}_{q}^{\prime}(\widehat{z})d\widehat{z}$$

$${E}_{fq}^{(3)}={\int }_{0}^{\infty }{\mathfrak{F}}_{f}(\widehat{z}){\mathfrak{H}}_{q}^{*\prime}(\widehat{z})d\widehat{z}{;F}_{ek}^{\left(1\right)}={\mathfrak{W}}_{5}{\int }_{0}^{\infty }{\mathfrak{Y}}_{e}(\widehat{r}){\mathfrak{I}}_{k}^{\prime}(\widehat{r})d\widehat{r};{F}_{fd}^{\left(2\right)}={\int }_{0}^{\infty }{\mathfrak{F}}_{f}(\widehat{z}){\upphi}_{d}(\widehat{z})d\widehat{z}$$

$${M}_{gp}^{(4)}={\int }_{0}^{\infty }{\mathfrak{R}}_{g}(\widehat{r}){\mathfrak{R}}_{p}(\widehat{r})d\widehat{r};{M}_{hq}^{(5)}={\int }_{0}^{\infty }{\mathfrak{H}}_{h}(\widehat{z}){\mathfrak{H}}_{q}(\widehat{z})d\widehat{z};{M}_{hq}^{(6)}={\int }_{0}^{\infty }{\mathfrak{H}}_{h}(\widehat{z}){\mathfrak{H}}_{q}^{*}(\widehat{z})d\widehat{z}$$

$${C}_{gp}^{(4)}={\mathfrak{W}}_{1}{\int }_{0}^{\infty }{\mathfrak{R}}_{g}(\widehat{r}){\mathfrak{R}}_{p}(\widehat{r})d\widehat{r};{C}_{hq}^{(5)}={\int }_{0}^{\infty }{\mathfrak{H}}_{h}(\widehat{z}){\mathfrak{H}}_{q}(\widehat{z})d\widehat{z};{C}_{hq}^{(6)}={\int }_{0}^{\infty }{\mathfrak{H}}_{h}(\widehat{z}){\mathfrak{H}}_{q}^{*}(\widehat{z})d\widehat{z}$$

$${E}_{gp}^{(4)}={\mathfrak{W}}_{2}{\int }_{0}^{\infty }{\mathfrak{R}}_{g}(\widehat{r}){\mathfrak{R}}_{p}\left(\widehat{r}\right)d\overline{r };{E}_{hq}^{(5)}={\int }_{0}^{\infty }{\mathfrak{H}}_{h}(\widehat{z}){\mathfrak{H}}_{q}^{\prime\prime}(\widehat{z})d\widehat{z};{E}_{hq}^{\left(6\right)}={\int }_{0}^{\infty }{\mathfrak{H}}_{h}(\widehat{z}){\mathfrak{H}}_{q}^{*\prime\prime}(\widehat{z})d\widehat{z}$$

$${E}_{gp}^{(7)}={\mathfrak{W}}_{3}{\int }_{0}^{\infty }{\mathfrak{R}}_{g}(\widehat{r})\left({\mathfrak{R}}_{p}^{\prime\prime}\left(\widehat{r}\right)+\frac{1}{\widehat{r}}{\mathfrak{R}}_{p}^{\prime}\left(\widehat{r}\right)\right)d\overline{r };{E}_{hq}^{(8)}={\int }_{0}^{\infty }{\mathfrak{H}}_{h}(\widehat{z}){\mathfrak{H}}_{q}(\widehat{z})d\widehat{z}$$

$${E}_{hq}^{\left(9\right)}={\int }_{0}^{\infty }{\mathfrak{H}}_{h}(\widehat{z}){\mathfrak{H}}_{q}^{*}(\widehat{z})d\widehat{z};{K}_{gi}^{\left(7\right)}={\mathfrak{W}}_{4}{\int }_{0}^{\infty }{\mathfrak{R}}_{g}(\widehat{r})\left({\mathfrak{Y}}_{i}^{\prime}\left(\widehat{r}\right)+\frac{1}{\overline{r}}{\mathfrak{Y} }_{i}\left(\widehat{r}\right)\right)d\widehat{r}$$

$${K}_{hj}^{\left(8\right)}={\int }_{0}^{\infty }{\mathfrak{H}}_{h}(\widehat{z}){\mathfrak{F}}_{j}^{\prime}(\widehat{z})d\widehat{z};{K}_{hj}^{\left(9\right)}={\int }_{0}^{\infty }{\mathfrak{H}}_{h}(\widehat{z}){\mathfrak{F}}_{j}^{*\prime}(\widehat{z})d\widehat{z};{F}_{gk}^{\left(3\right)}={\mathfrak{W}}_{5}{\int }_{0}^{\infty }{\mathfrak{R}}_{g}(\widehat{r}){\uppsi}_{k}(\widehat{r})d\widehat{r}$$

$${F}_{hd}^{\left(4\right)}={\int }_{0}^{\infty }{\mathfrak{H}}_{h}(\widehat{z}){\upphi}_{d}^{\prime}(\widehat{z})d\widehat{z};{M}_{ck}^{(7)}={\int }_{0}^{\infty }{\mathfrak{I}}_{c}(\widehat{r}){\mathfrak{I}}_{k}(\widehat{r})d\widehat{r};{M}_{ld}^{(8)}={\int }_{0}^{\infty }{\mathfrak{L}}_{l}\left(\widehat{z}\right){\upphi}_{l}\left(\widehat{z}\right)d\widehat{z};$$

$${M}_{ci}^{(9)}={\mathfrak{N}}_{1}{\int }_{0}^{\infty }{\mathfrak{I}}_{c}(\widehat{r})\left({\mathfrak{Y}}_{i}^{\prime}\left(\widehat{r}\right)+\frac{1}{\overline{r}}{\mathfrak{Y} }_{i}\left(\widehat{r}\right)\right)d\widehat{r};{M}_{lj}^{(10)}={\int }_{0}^{\infty }{\upphi}_{l}\left(\widehat{z}\right){\mathfrak{F}}_{j}\left(\widehat{z}\right)d\widehat{z};$$

$${M}_{lj}^{\left(11\right)}={\int }_{0}^{\infty }{\upphi}_{l}\left(\widehat{z}\right){\mathfrak{F}}_{j}^{*}\left(\widehat{z}\right)d\widehat{z};{M}_{cp}^{\left(12\right)}={\mathfrak{N}}_{1}{\int }_{0}^{\infty }{\mathfrak{I}}_{c}\left(\widehat{r}\right){\mathfrak{R}}_{p}\left(\widehat{r}\right)d\widehat{r};$$

$${M}_{lq}^{\left(13\right)}={\int }_{0}^{\infty }{\upphi}_{l}\left(\widehat{z}\right){\mathfrak{H}}_{q}^{\prime}\left(\widehat{z}\right)d\widehat{z};{M}_{lq}^{\left(14\right)}={\int }_{0}^{\infty }{\upphi}_{l}\left(\widehat{z}\right){\mathfrak{H}}_{q}^{*\prime}\left(\widehat{z}\right)d\widehat{z};{C}_{ck}^{\left(7\right)}={\mathfrak{N}}_{2}{\int }_{0}^{\infty }{\mathfrak{I}}_{c}\left(\overline{r }\right){\mathfrak{I}}_{k}\left(\overline{r }\right)d\widehat{r}$$

$${C}_{ld}^{\left(8\right)}={\int }_{0}^{\infty }{\upphi}_{l}\left(\widehat{z}\right){\upphi}_{l}\left(\widehat{z}\right)d\widehat{z};{C}_{ci}^{\left(9\right)}={\mathfrak{N}}_{3}{\int }_{0}^{\infty }{\mathfrak{I}}_{c}\left(\overline{r }\right)\left({\mathfrak{Y}}_{i}^{\prime}\left(\widehat{r}\right)+\frac{1}{\overline{r}}{\mathfrak{Y} }_{i}\left(\widehat{r}\right)\right)d\widehat{r};$$

$${C}_{lj}^{\left(10\right)}={\int }_{0}^{\infty }{\upphi}_{l}\left(\widehat{z}\right){\mathfrak{F}}_{j}\left(\widehat{z}\right)d\widehat{z};{C}_{lj}^{\left(11\right)}={\int }_{0}^{\infty }{\upphi}_{l}\left(\widehat{z}\right){\mathfrak{F}}_{j}^{*}\left(\widehat{z}\right)d\widehat{z};{C}_{cp}^{\left(12\right)}={\mathfrak{N}}_{3}{\int }_{0}^{\infty }{\mathfrak{I}}_{k}(\widehat{r}){\mathfrak{R}}_{p}(\widehat{r})d\widehat{r}$$

$${C}_{cp}^{\left(13\right)}={\int }_{0}^{\infty }{\mathfrak{L}}_{d}(\widehat{z}){\mathfrak{H}}_{q}^{\prime}\left(\widehat{z}\right)d\widehat{z};{C}_{cp}^{\left(14\right)}={\int }_{0}^{\infty }{\mathfrak{L}}_{d}(\widehat{z}){\mathfrak{H}}_{q}^{*\prime}\left(\widehat{z}\right)d\widehat{z};$$

$${F}_{ck}^{\left(5\right)}={\int }_{0}^{\infty }{{\mathfrak{N}}_{7}\mathfrak{I}}_{c}\left(\widehat{r}\right)\left(\left({\mathfrak{I}}_{k}^{\prime\prime}\left(\widehat{r}\right)+\frac{1}{r}{\mathfrak{I}}_{k}^{\prime}\left(\widehat{r}\right)\right)-\left({\mathfrak{N}}_{4}{\mathfrak{I}}_{k}\left(\widehat{r}\right)\right)\right)d\widehat{r};$$

$${F}_{ld}^{\left(6\right)}={\int }_{0}^{\infty }{\upphi}_{l}\left(\widehat{z}\right){\upphi}_{d}\left(\widehat{z}\right)d\widehat{z};$$

$${F}_{ck}^{\left(7\right)}={\mathfrak{N}}_{7}{\int }_{0}^{\infty }{\mathfrak{I}}_{c}\left(\widehat{r}\right){\mathfrak{I}}_{k}\left(\widehat{r}\right)d\widehat{r}{;F}_{ld}^{\left(8\right)}={\int }_{0}^{\infty }{\mathfrak{L}}_{l}\left(\widehat{z}\right){\mathfrak{L}}_{d}^{\prime\prime}\left(\widehat{z}\right)d\widehat{z};$$

$${P}_{cl}^{\left(1\right)}={\mathfrak{N}}_{4}{\widehat{T}}_{b}\left({\int }_{0}^{\infty }{\mathfrak{I}}_{c}\left(\widehat{r}\right)d\widehat{r}\times {\int }_{0}^{\infty }{\mathfrak{L}}_{l}\left(\widehat{z}\right)d\widehat{z};\right);$$

$${P}_{cl}^{\left(2\right)}\left(\widehat{t}\right)={\mathfrak{N}}_{5}\left({\int }_{0}^{\infty }{\mathfrak{I}}_{c}\left(\widehat{r}\right)d\widehat{r}\times {\int }_{0}^{\infty }{f}_{1}\left(\widehat{z}\right){\mathfrak{L}}_{l}\left(\widehat{z}\right)d\widehat{z};\right){\fancyscript{f}}_{2}\left(\widehat{t}\right);$$

$${P}_{cl}^{\left(3\right)}(\widehat{t})={\mathfrak{N}}_{6}\left({\int }_{0}^{\infty }{\mathfrak{I}}_{c}(\widehat{r})d\widehat{r}\times {\int }_{0}^{\infty }{f}_{1}(\widehat{z}){\mathfrak{L}}_{l}\left(\widehat{z}\right)d\widehat{z};\right){\dot{\fancyscript{f}}}_{2}(\widehat{t})$$

$${K}_{ei}^{(10)}={\int }_{0}^{\infty }{\mathfrak{Y}}_{e}(\widehat{r}){\mathfrak{Y}}_{i}\left(\widehat{r}\right)d\widehat{r}; {K}_{j}^{(11)}={\mathfrak{F}}_{j}^{\prime}\left(0\right);{K}_{j}^{(12)}={\mathfrak{F}}_{j}^{*\prime}\left(0\right);$$

$${E}_{ep}^{\left(10\right)}={\int }_{0}^{\infty }{\mathfrak{Y}}_{e}(\widehat{r}){\mathfrak{R}}_{p}^{\prime}\left(\widehat{r}\right)d\widehat{r};{E}_{q}^{\left(11\right)}={\mathfrak{H}}_{q}\left(0\right);{E}_{q}^{\left(12\right)}={\mathfrak{H}}_{q}^{*}\left(0\right);$$

$${K}_{gi}^{(13)}={\int }_{0}^{\infty }{\mathfrak{R}}_{g}(\widehat{r})\left({\mathfrak{Y}}_{i}^{\prime}\left(\widehat{r}\right)+\frac{1}{\overline{r}}{\mathfrak{Y} }_{i}\left(\widehat{r}\right)\right)d\widehat{r};{K}_{gi}^{(14)}= {\mathfrak{F}}_{j}\left(0\right);{K}_{j}^{(15)}={\mathfrak{F}}_{j}^{*}\left(0\right);$$

$${E}_{gp}^{\left(13\right)}={\mathfrak{M}}_{6}{\int }_{0}^{\infty }{\mathfrak{R}}_{g}\left(\widehat{r}\right){\mathfrak{R}}_{p}\left(\widehat{r}\right)d\widehat{r};{E}_{gp}^{\left(14\right)}={\mathfrak{H}}_{q}^{\prime}\left(0\right);{E}_{q}^{\left(15\right)}={\mathfrak{H}}_{q}^{*\prime}\left(0\right);$$

$${F}_{gk}^{\left(9\right)}={\mathfrak{M}}_{7}{\int }_{0}^{\infty }{\mathfrak{R}}_{g}\left(\widehat{r}\right){\mathfrak{I}}_{k}\left(\widehat{r}\right)d\widehat{r};{F}_{D}^{\left(10\right)}={\mathfrak{L}}_{l}(0)$$

## Appendix B

$${\mathcal{M}}_{11}^{\left(1\right)}={M}_{11}^{\left(1\right)}{M}_{11}^{\left(2\right)};{\mathcal{M}}_{11}^{\left(2\right)}={\mathfrak{d}}_{1}{M}_{11}^{\left(1\right)}{M}_{11}^{\left(3\right)};{\mathcal{C}}_{11}^{\left(1\right)}={C}_{11}^{\left(1\right)}{C}_{11}^{\left(2\right)};{\mathcal{C}}_{11}^{\left(2\right)}={\mathfrak{d}}_{1}{C}_{11}^{\left(1\right)}{C}_{11}^{\left(3\right)};$$

$${\mathcal{K}}_{11}^{(1)}={K}_{11}^{(1)}{K}_{11}^{(2)}+{K}_{11}^{(4)}{K}_{11}^{(5)}+{\mathfrak{d}}_{2}{E}_{11}^{(1)}{E}_{11}^{(3)};$$

$${\mathcal{K}}_{11}^{(2)}={\mathfrak{d}}_{1}\left({K}_{11}^{(1)}{K}_{11}^{(3)}+{K}_{11}^{(4)}{K}_{11}^{(6)}\right)+\left({E}_{11}^{(1)}{E}_{11}^{(2)}\right);{\mathcal{K}}_{11}^{(3)}={F}_{11}^{(1)}{F}_{11}^{(2)}-{\mathfrak{d}}_{3}{E}_{11}^{(1)}{E}_{11}^{(3)}$$

$${\mathcal{M}}_{11}^{(3)}={M}_{11}^{(4)}{M}_{11}^{(5)};{\mathcal{M}}_{11}^{(4)}={\mathfrak{d}}_{2}{M}_{11}^{(4)}{M}_{11}^{(6)};{\mathcal{M}}_{11}^{(5)}={\mathfrak{d}}_{3}{M}_{11}^{(4)}{M}_{11}^{(6)}$$

$${\mathcal{C}}_{11}^{(3)}={C}_{11}^{(4)}{C}_{11}^{(5)};{\mathcal{M}}_{11}^{(4)}={\mathfrak{d}}_{2}{C}_{11}^{(4)}{C}_{11}^{(6)};{\mathcal{C}}_{11}^{(5)}={\mathfrak{d}}_{3}{C}_{11}^{(4)}{C}_{11}^{(6)}$$

$${\mathcal{K}}_{11}^{(4)}=\left({E}_{11}^{(4)}{E}_{11}^{(5)}+{E}_{11}^{(7)}{E}_{11}^{(8)}\right)+{\mathfrak{d}}_{1}{K}_{11}^{(7)}{K}_{11}^{(9)}$$

$${\mathcal{K}}_{11}^{\left(5\right)}={\mathfrak{d}}_{2}\left({E}_{11}^{\left(4\right)}{E}_{11}^{\left(6\right)}+{E}_{11}^{\left(7\right)}{E}_{11}^{\left(9\right)}\right)+{K}_{11}^{\left(7\right)}{K}_{11}^{\left(8\right)};$$

$${\mathcal{K}}_{11}^{(5)}={F}_{11}^{(3)}{F}_{11}^{(3)}-{\mathfrak{d}}_{3}\left({E}_{11}^{\left(4\right)}{E}_{11}^{\left(6\right)}+{E}_{11}^{\left(7\right)}{E}_{11}^{\left(9\right)}\right);{\mathcal{M}}_{11}^{\left(6\right)}={M}_{11}^{\left(7\right)}{M}_{11}^{\left(8\right)}+{\mathfrak{d}}_{3}{M}_{11}^{\left(12\right)}{M}_{11}^{\left(14\right)};$$

$${\mathcal{M}}_{11}^{\left(7\right)}={M}_{11}^{\left(9\right)}{M}_{11}^{\left(10\right)}+{\mathfrak{d}}_{2}{M}_{11}^{\left(12\right)}{M}_{11}^{\left(14\right)};{\mathcal{M}}_{11}^{\left(8\right)}={\mathfrak{d}}_{1}{M}_{11}^{\left(9\right)}{M}_{11}^{\left(11\right)}+{M}_{11}^{\left(12\right)}{M}_{11}^{\left(13\right)};$$

$${\mathcal{C}}_{11}^{(6)}={C}_{11}^{\left(7\right)}{C}_{11}^{\left(8\right)}+{\mathfrak{d}}_{3}{C}_{11}^{\left(12\right)}{C}_{11}^{\left(14\right)};{\mathcal{C}}_{11}^{(7)}={C}_{11}^{\left(9\right)}{C}_{11}^{\left(10\right)}+{\mathfrak{d}}_{2}{C}_{11}^{\left(12\right)}{C}_{11}^{\left(14\right)};$$

$${\mathcal{C}}_{11}^{(8)}={C}_{11}^{\left(12\right)}{C}_{11}^{\left(13\right)}+{\mathfrak{d}}_{1}{C}_{11}^{\left(9\right)}{C}_{11}^{\left(11\right)};{\mathcal{K}}^{(7)}={F}_{11}^{\left(5\right)}{F}_{11}^{\left(6\right)}+{F}_{11}^{\left(7\right)}{F}_{11}^{\left(8\right)}$$
